# Personalized Physician-Assisted Sleep Advice for Shift Workers: Algorithm Development and Validation Study

**DOI:** 10.2196/65000

**Published:** 2025-04-01

**Authors:** Yufei Shen, Alicia Choto Olivier, Han Yu, Asami Ito-Masui, Ryota Sakamoto, Motomu Shimaoka, Akane Sano

**Affiliations:** 1 Rice University Houston, TX United States; 2 The University of Texas at Austin Austin, TX United States; 3 Mie University Tsu Japan

**Keywords:** cognitive behavioral therapy, CBT, health care workers, machine learning, medical safety, web-based intervention, app-based intervention, shift work, shift work sleep disorders, shift workers, sleep disorder, wearable sensors, well-being

## Abstract

**Background:**

In the modern economy, shift work is prevalent in numerous occupations. However, it often disrupts workers’ circadian rhythms and can result in shift work sleep disorder. Proper management of shift work sleep disorder involves comprehensive and patient-specific strategies, some of which are similar to cognitive behavioral therapy for insomnia.

**Objective:**

Our goal was to develop and evaluate machine learning algorithms that predict physicians’ sleep advice using wearable and survey data. We developed a web- and app-based system to provide individualized sleep and behavior advice based on cognitive behavioral therapy for insomnia for shift workers.

**Methods:**

Data were collected for 5 weeks from shift workers (N=61) in the intensive care unit at 2 hospitals in Japan. The data comprised 3 modalities: Fitbit data, survey data, and sleep advice. After the first week of enrollment, physicians reviewed Fitbit and survey data to provide sleep advice and selected 1 to 5 messages from a list of 23 options. We handcrafted physiological and behavioral features from the raw data and identified clusters of participants with similar characteristics using hierarchical clustering. We explored 3 models (random forest, light gradient-boosting machine, and CatBoost) and 3 data-balancing approaches (no balancing, random oversampling, and synthetic minority oversampling technique) to predict selections for the 7 most frequent advice messages related to bedroom brightness, smartphone use, and nap and sleep duration. We tested our predictions under participant-dependent and participant-independent settings and analyzed the most important features for prediction using permutation importance and Shapley additive explanations.

**Results:**

We found that the clusters were distinguished by work shifts and behavioral patterns. For example, one cluster had days with low sleep duration and the lowest sleep quality when there was a day shift on the day before and a midnight shift on the current day. Our advice prediction models achieved a higher area under the precision-recall curve than the baseline in all settings. The performance differences were statistically significant (*P*<.001 for 13 tests and *P*=.003 for 1 test). Sensitivity ranged from 0.50 to 1.00, and specificity varied between 0.44 and 0.93 across all advice messages and dataset split settings. Feature importance analysis of our models found several important features that matched the corresponding advice messages sent. For instance, for message 7 (*darken the bedroom when you go to bed*), the models primarily examined the average brightness of the sleep environment to make predictions.

**Conclusions:**

Although our current system requires physician input, an accurate machine learning algorithm shows promise for automatic advice without compromising the trustworthiness of the selected recommendations. Despite its decent performance, the algorithm is currently limited to the 7 most popular messages. Further studies are needed to enable predictions for less frequent advice labels.

## Introduction

### Background

In the modern economy, shift work is prevalent in numerous occupations. Data from 2010 estimated that half of the workers in food preparation and serving, >45% of workers in protective service, and >35% of health care practitioners had alternative shifts apart from regular day shifts in the United States [1]. However, shift work often conflicts with the daily rhythm of sleep and wakefulness, which increases the risk of shift work sleep disorder (SWSD), a circadian rhythm disorder characterized by shift work–related sleepiness and insomnia [2]. Multiple studies have shown the substantial prevalence of SWSD among shift workers. A survey of nurses in 3 federal hospitals in Ethiopia found that 25.6% (102/399) of participants had SWSD [3]. Another study of nurses in Norway reported prevalence rates of 44.2% (580/1313) and 23.6% (146/619) for SWSD-indicative symptoms among night workers and day workers, respectively [4]. A random population sample of 1163 participants in Australia revealed that 10.1% (91/898) of day workers and 32.1% (85/265) of night workers fulfilled the study’s criteria for SWSD and 1.3% (12/898) of day workers and 9.1% (24/265) of night workers were further classified under severe SWSD based on the extent of negative SWSD impacts on their life [5]. Shift workers’ sleep problems are also associated with risks of mental health issues [6-8], work errors [8], burnout [6], and turnover intentions [9].

Proper management of SWSD emphasizes comprehensive and patient-specific strategies such as circadian adaptation, maintaining good sleep hygiene, strategic napping, clockwise shift rotation, and wakefulness promotions for night shifts [10]. Some of these strategies are analogous to the cognitive behavioral therapy for insomnia (CBTI), a psychological therapy recommended by the American College of Physicians as initial treatment for chronic insomnia [11], which consists of multiple approaches to improve sleep, including education about normal sleep, sleep hygiene practice, sleep restriction to consolidate high-quality sleep, and relaxation [12].

### Related Work

Researchers have experimented with CBTI on shift workers with sleep problems and high risk of SWSD. A study by Järnefelt et al [13] on 19 shift workers with insomnia, more than half of whom exhibited SWSD characteristics, showed that group-based CBTI treatment improved their insomnia severity and sleep over a 6-month postintervention period, and a follow-up study further demonstrated long-lasting benefits of CBTI for up to 24 months after the intervention [14]. Järnefelt et al [15] also conducted a randomized controlled trial of group-based CBTI and self-help CBTI against a sleep hygiene education control on 83 shift workers with insomnia more recently. They found that group-based CBTI improved participants’ mental health significantly, although both CBTI interventions enhanced their sleep and reduced their insomnia symptoms without significant differences from the control group, and all interventions were more beneficial for participants without SWSD characteristics [15]. Peter et al [16] used web-based CBTI for shift workers with sleep problems (n=21) and found it to be as effective as face-to-face SWSD outpatient CBTI treatment (n=12) for improving sleep efficiency and insomnia symptoms. Their research group also proposed a randomized controlled trial in 2021 to further assess their web-based CBTI intervention for shift workers against a waitlist control and face-to-face CBTI [17]. Although this existing research on CBTI for shift workers has shown promising improvement in participants’ sleep, group-based and face-to-face CBTI required participants to attend several weekly sessions for up to 120 minutes led by specialists trained for >10 hours, and the long time commitment likely caused many participants to miss some sessions, which might have weakened the intervention effects [13,15,16]. Self-help, web-based CBTI was relatively more accessible to shift workers, but time commitment for weekly sessions was still necessary and likely contributed to the considerable dropout rates, and the interventions either required human input for professional feedback or only resembled a web-based learning module without any personalization [15,16]. Therefore, a system that automatically provides personalized CBTI interventions and sleep recommendations for shift workers and requires less time commitment could benefit them substantially and yield low dropout rates.

In recent years, several web-based and mobile CBTI systems have been developed to deliver automated and individualized CBTI interventions. For example, Sleep Healthy Using the Internet [18] and Sleepio [19] provide automated web-based CBTI modules equipped with interactive web design elements and user-tailored recommendations, and multiple randomized controlled trials have demonstrated their effectiveness against insomnia and related mental health problems among various demographic groups [18,20-23]. Beun et al [24,25] developed Sleepcare, a mobile app equipped with an automated digital coach interacting with users to provide CBTI interventions, adapt interventions to user characteristics, and promote adherence. Its efficacy has been demonstrated in a randomized controlled trial of 151 participants by Horsch et al [26]. CBT-i Coach is another phone app developed as a supplemental tool for face-to-face CBTI in which users can receive sleep education, customize sleep hygiene advice and relaxation activities, and practice personalized sleep restrictions [27]. In a pilot study, the app did not impair CBTI effectiveness and potentially improved patient adherence [28]. A subsequent variant of the app called Insomnia Coach was applied as a self-help CBTI intervention on 25 veterans with insomnia and showed significant treatment effects compared to the waitlist control [29].

In addition to web-based and mobile CBTI, multiple automated sleep recommender systems (RSs) have also been developed to provide personalized sleep suggestions. SleepCoacher collects data from smartphone sensors and user self-reports to compute sleep variables and includes recommendation templates reviewed by clinicians for each variable combination [30]. To deliver a recommendation, the system chooses the template of the most correlated combination, further personalizes it according to the user’s sleep status, and also evaluates the effectiveness of the recommendation. In total, 2 studies on 43 undergraduate students showed the benefits of this system on sleep, which were greater when users adhered more to the system. Daskalova et al [31] explored cohort-based sleep recommendations in which their system used demographic, exercise, and sleep data collected from 1 million users of a wearable device to assign each participant to a user group in which all members had similar profiles and chose a recommendation that showed the greatest improvement among the group in some aspects of sleep behaviors on which the participant performed poorly compared to these similar users. Compared to general sleep suggestions, cohort-based recommendations improved sleep duration more, and many participants were motivated to follow these recommendations. Pandey et al [32] used event mining to discover optimal causal relationships between a user’s lifestyle and sleep and provided recommendations to match their current behaviors and environment with these relationships for better sleep. On the other hand, some systems do not directly provide advice to users but, instead, help them self-adjust their sleep habits. For example, Lullaby uses multiple sensors to track users’ sleep and environment and helps them discover environmental factors with negative impacts on their sleep [33]. ShutEye serves as a peripheral display on mobile phones to remind users of recommended and inadvisable time windows for various sleep-related activities [34]. SleepBandits enables users to perform numerous self-experiments to adjust their behaviors and uses data from mobile phone sensors to evaluate experiment effects on their sleep [35].

Although existing automated and individualized CBTI and sleep RSs have demonstrated their great potential in improving sleep, they have not been modified to accommodate shift workers’ irregular schedules of shift and wakefulness [13] and, thus, do not include strategies specifically beneficial to shift workers. Moreover, these CBTI interventions are mostly delivered through weekly learning modules and tasks requiring active involvement and a regular time commitment, and their personalization options are limited and mainly focus on sleep restrictions. Unlike CBTI, sleep RSs directly send behavior change recommendations to users, but their advice-tailoring processes often lack clinical support and have raised doubts about their credibility among users [31].

### Objectives

We aimed to develop and evaluate a machine learning algorithm that predicts physicians’ sleep advice using wearable and survey data. We also examined the model’s potential in assisting physicians and even replacing them in providing sleep advice in the system.

We characterized shift workers’ behaviors using clustering analyses, estimated physicians’ advice selection strategies by interpreting the developed advice prediction models, and assessed the effectiveness of the provided sleep recommendations to gain more insights into the system.

## Methods

### Data Collection

The data were collected from shift workers, including physicians and nurses, in the intensive care unit (ICU) at Mie University Hospital (n=47) and Suzuka Chuo General Hospital (n=14) in Japan [36,37]. Eligible participants must have 8 hours of work per shift and poor sleep quality demonstrated through a Pittsburgh Sleep Quality Index score of ≥5. The detailed inclusion and exclusion criteria have been described in the work by Ito-Masui et al [36,37]. Each participant enrolled in a 5-week trial, with the first week as a baseline without an intervention (preintervention time point) and the following 4 weeks with interventions. During the 5 weeks, participants filled out daily surveys and wore a Fitbit (Google) to collect physiological and behavioral data. Physicians reviewed the data and provided sleep advice to participants as interventions 3 to 4 times a week. In total, 2123 days of multimodal data were collected from these participants for the entire study.

### Ethical Considerations

This study was approved by the Clinical Research Ethics Review Committee of Mie University Hospital, Tsu, Japan (H2020-083). This study was approved by the ethics committee of Suzuka General Hospital (review number 254). All procedures were performed in accordance with the ethical standards of the institutional research committee and the 2013 Declaration of Helsinki. This study was registered in the University Hospital Medical Information Network Clinical Trials Registry on May 1, 2020 (ID: UMIN000040547). Participants provided written consent after receiving sufficient information about the study. They retained the right to opt out of the study at any time. To protect privacy, all collected data were deidentified. No compensation was offered for participation.

### Data Modalities

The collected data included 3 main modalities—Fitbit data, survey data, and sleep advice. We followed the methods by Yu et al [38] to extract some features from the same Fitbit and survey data, and engineered extra features to accommodate our objectives.

#### Fitbit Data

Raw data from Fitbit included minute-by-minute steps and heart rate as well as the start time, end time, duration, and efficiency of each sleep period. From raw heart rate data, we computed their average, SD, and sample entropy. Sample entropy has been used for cardiovascular time series, and a low entropy value suggests greater self-similarity of the series [39]. We also computed features from step count per minute to account for the variability in participants’ daily activities. Specifically, we computed and stored the duration of each continuous segment with and without steps in minutes. We then computed information entropy for the stationary and active segments. A higher entropy corresponded to more variability in participants’ moving or nonmoving behaviors. Finally, we retrieved information about the main sleep period with the longest duration from raw sleep data.

#### Survey Data

Participants received a morning survey and an evening survey every day with questions about their daily behaviors and well-being. Features were then extracted from their answers.

##### Morning Survey

Features from the morning surveys were divided into 3 categories—sleep, well-being, and miscellany.

###### Sleep

In the morning surveys, participants indicated whether they had slept in the previous 24 hours and provided the number of naps between 8 AM and 8 AM on the following day. They also described how they woke up (naturally, via an alarm, or via other means) and reported the time it took for them to fall asleep, the duration of their phone use before sleep, the brightness level of their sleep environment, and sleep quality. Specifically, sleep quality was quantified through the answers to the following statements using 5-point Likert scales (*strongly disagree*=1; *strongly agree*=5): (1) *I slept soundly*, (2) *I fell asleep immediately*, (3) *I was able to recover from fatigue*, (4) *I didn’t wake up in the middle of sleep*, and (5) *I was satisfied with sleep*.

In addition to the aforementioned features retrieved directly from surveys, we constructed some sleep features using available survey data. In the surveys, participants reported the start time and end time of their main sleep and nap periods. According to the time entries, we constructed a binary sequence with 1-minute resolution for every day, in which a bit of 1 meant that the participants were sleeping or napping at that minute of the day. If time entries of the main sleep periods were missing from the surveys, the entries from Fitbit were used. From the binary sequences, we computed the duration of the main sleep and nap periods. We also calculated the sleep regularity index (SRI) with sliding windows of 3, 5, and 7 days. The SRI represents the probability of any 2 time points that are 24 hours apart having the same sleep or awake state averaged over a specified time window and is scaled to range from −100 to 100, where a value of 100 indicates the same sleep schedule across all days and a value of −100 means that an individual has completely flipped their sleep schedule between any 2 consecutive days [40]. Previous studies have shown that the SRI is positively correlated with the academic performance of college students [40] and a lower SRI is associated with increased stress, depression, and risk of cardiovascular diseases in older adults [41].

###### Well-Being

Participants reported 6 well-being metrics in the morning surveys. A total of 5 metrics—alertness, happiness, energy, health, and calmness—were reported using a continuous scale from 1 to 100, with 100 as the most positive. The sixth metric, current sleepiness, was reported via a 9-point Likert scale from *strongly awake* (1) to *strongly sleepy* (9).

###### Behavior

The morning surveys also recorded participants’ daily behaviors, including the amount of caffeine intake and last intake time, amount of alcohol intake, and bath time. From the time entries, we computed the durations between their last caffeine intake and sleep and between bath and sleep.

##### Evening Survey

Features from the evening surveys were divided into 2 categories—work and well-being.

###### Work

In the evening surveys, participants reported their work schedules for the current day, the day before, and the day before the day before in binary sequences with 30-minute resolution, where a bit of 1 meant work during that 30-minute interval. From the binary sequences, we computed work hours for the entire day as well as for 3 different periods of the day (1: midnight-8 AM, 2: 8 AM-4 PM, and 3: 4 PM-midnight). The periods corresponded to midnight shifts, day shifts, and afternoon shifts of the participants. Moreover, by comparing the binary work sequences and binary sleep sequences constructed from the morning surveys, we calculated participants’ nap durations during work and minimum durations between the last sleep periods and the start of shifts and between the end of shifts and following sleep periods. A binary indicator of whether participants did any activities other than work outside their homes was also retrieved from the surveys.

###### Well-Being

Similarly, as in the morning surveys, participants also answered several questions about their well-being in the evening surveys. In addition to the 6 metrics reported in the morning surveys (alertness, happiness, energy, health, calmness, and current sleepiness), they provided 3 other metrics: sleepiness during the day, stress, and tiredness, which were all reported via 5-point Likert scales (5=*strongly awake*, *stressful*, or *tired*).

#### Sleep Advice

Starting from the second week of enrollment, physicians reviewed Fitbit and survey data to provide sleep advice. One physician provided advice once a week to all participants, and 2 physicians gave advice 2 to 3 times a week to participants from each of the 2 hospitals. Over the 4 weeks with interventions, participants received advice approximately 13 times, and a total of 786 pieces of advice were sent to the 61 participants who completed their 5 weeks of the study. Every time physicians provided sleep advice, they chose 1 to 5 messages from 23 options, listed in Table 1. To better summarize the options, we divided them into 6 categories according to their descriptions: (1) dietary intake (messages 1, 2, and 3), (2) activity (messages 4, 5, 6, 16, 17, and 18), (3) sleep (messages 7, 8, 9, 10, 20, 21, and 22), (4) shift (message 11), (5) nap (messages 12, 13, 14, 15, and 23), and (6) mentality (messages 19). After participants received the sleep advice, they were able to respond to the advice as *eager to follow* or *difficult to follow*.

**Table 1:** Content of the 23 sleep advice messages that physicians sent to the participants.

Message category and ID		Message content
**Dietary intake**		
	1	“Refrain from consuming alcohol before sleep.”
	2	“Stop consuming caffeine 3 hours before lights-out.”
	3	“Refrain from eating midnight snacks.”
**Activity**		
	4	“Refrain from using a smartphone in the bedroom.”
	5	“Take a bath a little earlier than usual.”
	6	“Refrain from exercising 3 hours before lights-out.”
	16	“Relax. 1) Make a habit of relaxing before sleep, including taking a bath in warm water, light stretching, using aroma, and drinking herbal tea.”
	17	“Relax. 2) Use the Fitbit breathing program called Relax.”
	18	“Do moderate exercise regularly.”
**Sleep**		
	7	“Darken the bedroom when you go to bed.”
	8	“When you do not fall asleep within 15 minutes, leave the bedroom and stay out of the bedroom until you get sleepy.”
	9	“Set your wake-up time according to each work shift.”
	10	“Do not try too hard to fall asleep quickly.”
	20	“Continue current sleep habits.”
	21	“Try to make enough time for sleep.”
	22	“Get up at the same time, whether you are working or on holiday.”
**Shift**		
	11	“Management of work shift: Consult with your superior so that shift rotation will be clockwise in general.”
**Nap**		
	12	“Take a nap. 1) If possible, take a nap for approximately 90 minutes before the night shift.”
	13	“Take a nap. 2) If possible, take a nap for 15–20 minutes during rest time while you are working.”
	14	“Take a nap. 3) If possible, take a nap earlier after a late-night shift to refresh.”
	15	“Create an environment for taking a nap: Ask your family for cooperation to create a quiet environment during a nap in the daytime or evening at home. Sharing your work shift schedule with your family by placing it where every member of your family can see it might be a good idea. Avoid strong lights for several hours before a nap and darken the room during a nap.”
	23	“Do not nap for too long.”
**Mentality**		
	19	“Be broad-minded and try to approach things positively.”

### Participant Characteristic Analyses

We investigated the characteristics of the participants’ daily activities to find participant subgroups who had similar behaviors and understand the relationship between their behaviors and well-being. We used agglomerative hierarchical clustering to find the groupings. The algorithm initiates all samples as individual clusters and merges clusters using Ward linkage to minimize the within-cluster variances at every step [42,43]. After all the samples are merged, a dendrogram is constructed to show the merged path for each sample. By setting thresholds on the height of the dendrogram, clusters and their samples can be identified.

We used daily features from Fitbit and the surveys, except for the participants’ well-being and sleep quality metrics, to find the clusters. To include as many samples as possible during clustering, we did not use the following features as they were not available for some days: SRI; nap duration during work; and the time between caffeine intake and sleep, between bath and sleep, between last sleep and the start of shift, and between the end of shift and following sleep (eg, participants did not work or take a bath). All features were standardized across all participants for clustering.

We then performed principal-component analysis on the standardized features and overlaid the cluster assignments on a 2D t-distributed stochastic neighbor embedding (t-SNE) plot generated from the principal components that explain 95% of the total variance to observe how clusters interacted with each other. t-SNE creates a low-dimensional mapping of high-dimensional data by matching the conditional probabilities in both spaces that are proportional to the similarity of the data points [44]. To characterize participants’ behaviors and well-being, we summarized the clusters’ profiles by observing feature averages and examined distributions of the well-being metrics for each cluster.

### Advice Prediction Models

As mentioned in the *Sleep Advice* section, physicians reviewed the Fitbit and survey data to provide sleep advice to participants. We developed machine learning models to predict their message selections and evaluated the performance of the advice prediction models.

#### Prediction Approach

The advice prediction task can be formulated as a binary classification, with the positive class representing the selection of an advice message. We found substantial class imbalances for the selected messages. Specifically, of 786 advice pieces, the number of selections ranged from 64 (8.1%) to 390 (49.6%) for the 7 most frequent advice messages (messages 4, 7, 12, 14, 15, 20, and 21). Consequently, we decided to build individual binary classifiers only for these messages as there would be too few positive samples to develop models for less frequent advice.

To construct samples for the machine learning models, we computed the average and SD of daily Fitbit and survey features across the previous 4 days for each date on which physicians provided advice. Samples with missing values were excluded. According to the physicians, they often considered the participants’ responses to previous advice, especially when they found it difficult to choose the messages. Therefore, we incorporated advice responses into our models and enabled the models to output class probabilities to indicate certainty about a message selection. We used 12 Fitbit features (steps, sleep, and heart rate), 53 morning survey features, 50 evening survey features, and 23 advice response features. Table S1 in Multimedia Appendix 1** **summarizes the features used by the classifier.

#### Dataset Split

To evaluate the advice prediction models, the data needed to be split into a training set and a test set. We designed 2 ways to split the data: a user-dependent split and a user-independent split. Under the user-dependent setting, the data were split chronologically, where the training set contained the first 70% of each participant’s data and the testing set had the remaining 30%. Under the user-independent setting, we split the data according to the order of participant enrollment such that the models were trained on data from the first 70% of participants enrolled and tested on the 30% of participants who enrolled later in the study. We chose these train-test split methods to ensure that, when predicting sleep advice for a date of a participant, no data from later days of this participant’s enrollment would be used in dependent models and only data from participants enrolled earlier would be used in independent models. In particular, the dependent split examined the effects of personalization by leveraging participants’ past data to predict their future sleep advice, whereas the independent split assessed model generalizability by predicting sleep messages for unseen participants.

#### Classifiers

Considering the abundant features but relatively small size of our data, we focused on ensemble classifiers for their feature selection capabilities and robustness to overfitting. In particular, we chose random forest (RF), a bagging-based model, and 2 gradient-boosting models, namely, light gradient-boosting machine (LGBM) [45] and CatBoost [46], as our candidates. To find the optimal sets of model hyperparameters, we tuned the classifiers on the training set using 5-fold stratified cross-validation and 100 iterations of random search over a grid of parameters. Random search was used instead of grid search because it can span a larger parameter space and find models as good as or better than those found using grid search under the same computation budget [47]. When tuning the classifiers under the user-independent setting, we made sure that the training data were divided into subsets with nonoverlapping participants. Finally, classifiers with optimal hyperparameters were fitted on the entire training set.

#### Data Balancing

To address class imbalances that could hinder the development of effective advice prediction models, we experimented with 2 data-balancing methods: random oversampling and synthetic minority oversampling technique (SMOTE) [48]. Random oversampling increases the representation of the minority class by duplicating existing samples through sampling with replacement. In contrast, SMOTE enhances the minority class by generating synthetic data points through interpolation between existing samples and their nearest neighbors. Data balancing was only applied to the training data during classifier tuning and training. To better understand the effectiveness of data balancing, we also trained models on the original imbalanced data for comparison.

#### Prediction Evaluation

By combining 3 classifiers with 3 data-balancing approaches, we trained 9 model configurations to predict the selection of a particular advice message under user-dependent and user-independent settings. On the basis of 2 key considerations, we chose the area under the precision-recall curve (AUPRC) as the main metric to evaluate and compare the performance of the different models. First, given the relatively few occurrences of sleep advice, it was crucial for the model to provide advice when a participant needed it. The precision-recall curve is particularly well suited for this scenario because it focuses on the model’s ability to correctly predict the positive class, which is actionable sleep advice. Second, due to class imbalances, the default decision threshold of 0.5 may not be optimal for converting the model’s probability outputs into predicted classes. As the precision-recall curve reflects the trade-off between false alarms and missed advice predictions across all decision thresholds, the AUPRC provides a comprehensive evaluation of the model’s ability to predict sleep advice without requiring a specific decision threshold.

For each model configuration, we repeated the tuning and training process 10 times with different random seeds and computed the average AUPRC on the test set. To ensure a fair comparison, the same set of seeds was used across all configurations. In addition, we performed 10 random guessing trials by sampling probability predictions from a standard uniform distribution and used a 1-sided Welch *t* test to assess whether the model performance significantly exceeded random guessing. This procedure was repeated for each of the 7 most frequent advice messages.

#### Feature Importance

After training the advice prediction models, we analyzed feature importance using 2 approaches to identify the key contributors to predictions on the test set. The first approach was permutation importance, which evaluates feature importance by measuring the decrease in prediction performance after randomly permuting a specific feature [49]. Greater performance degradation indicates higher feature importance. Using the AUPRC as the performance metric, we permuted each feature 10 times per classifier run and summarized the results on the test set. The second method was Shapley additive explanations (SHAP) [50,51], which leverages game theory to explain outputs of machine learning models at both local and global levels. In particular, we computed the mean absolute SHAP values across all test samples per classifier run to determine global feature importance. Both permutation and SHAP importance values were reported as averages across all repeated runs of each model configuration.

## Results

### Participant Characteristics

Tables 2 and 3 summarize the participants’ daily features and well-being scores, respectively. Their average step count was 7545 (SD 4419), their average sleep duration was 6.77 (SD 2.87) hours, their average nap duration was 53 (SD 108.2) minutes, their average sleep regularity was 52 (SD 19.3), and their average work duration was 5.62 (SD 4.19) hours.

**Figure 1:**
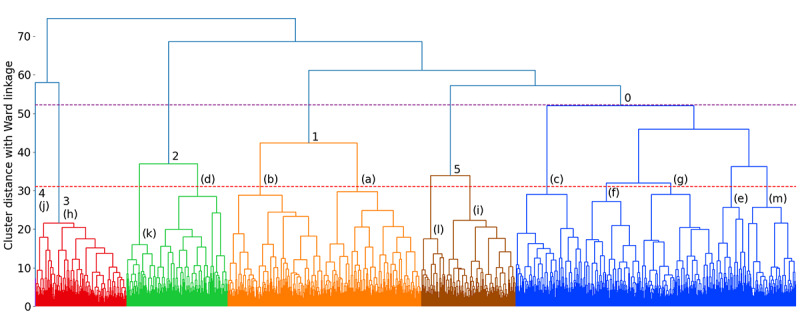
Dendrogram from hierarchical clustering of daily features using Ward linkage. The purple dashed line corresponds to a distance threshold of 52.24 (0.7 times the maximum cluster distance as implemented by SciPy [52]) and identifies 6 clusters labeled and color coded (cluster 0=blue, cluster 1=orange, cluster 2=green, cluster 3=red, cluster 4=purple, and cluster 5=brown). The red dashed line represents a distance threshold of 31 and identifies 13 subclusters (a-m).

We used 77.01% (1635/2123) of the samples with all selected features available for hierarchical clustering. Figure 1 shows the resulting dendrogram. The default distance threshold of 52.24 (purple dashed line) identified 6 clusters color coded and labeled with numbers from 0 to 5 in the dendrogram. In addition, a few smaller clusters merged at approximately 30, so we used a lower threshold of 31 (red dashed line) to divide the 6 clusters into 13 subclusters (*a*-*m*) and further investigated the behavior variability within each larger cluster. Specifically, cluster 0 had 5 subclusters: *c*, *f*, *g*, *e*, and *m*. Cluster 1 had 2 subclusters: *a* and *b*. Cluster 2 had 2 subclusters: *k* and *d*. Clusters 3 and 4 did not have any subclusters and themselves were labeled as *j* and *h*, respectively. Cluster 5 had 2 subclusters: *l* and *i*. Figure S1 in Multimedia Appendix 1 illustrates the clusters and subclusters overlaid on t-SNE plots obtained from the same data used for clustering. Cluster and subcluster structures were clearly observable in both plots. For example, cluster 0 in the plot of 6 clusters has an upper branch and a lower branch on either side of cluster 1, which are further divided into subclusters *c* and *g*, respectively, in the plot of 13 subclusters.

Table S2 in Multimedia Appendix 1 summarizes cluster and subcluster profiles for different aspects of participants’ behaviors and well-being. Major differences among the 6 clusters were observed in the well-being metrics, whereas subclusters can also be distinguished by work duration and shift types. The profiles will be discussed in detail later in the *Discussion* section.

**Table 2:** Participants’ daily physiological and behavioral features.

Feature	Values
hrmean^a^ (bpm), mean (SD)	74.87 (8.6)
hrstd^b^ (bpm), mean (SD)	13.42 (3.45)
hrentropy^c^, mean (SD)	0.63 (0.21)
steps, mean (SD)	7545 (4419.36)
duration_entropy_non_step, mean (SD)	2.34 (0.34)
duration_entropy_step, mean (SD)	1.69 (0.32)
nap_count, mean (SD)	0.56 (0.69)
brightness_sleep (scale of 1-100), mean (SD)	18.63 (23.09)
sleep_duration (min), mean (SD)	406.46 (172.01)
nap_duration (min), mean (SD)	52.99 (108.23)
sleep_regularity_3 days (scale of –100 to 100), mean (SD)	52.25 (28.3)
sleep_regularity_5 days (scale of –100 to 100), mean (SD)	52.1 (22.6)
sleep_regularity_7 days (scale of –100 to 100), mean (SD)	52.02 (19.26)
deep_sleep (scale of 1-5), mean (SD)	3.2 (1.14)
immediate_sleep (scale of 1-5), mean (SD)	3.55 (1.14)
fatigue_recover (scale of 1-5), mean (SD)	2.84 (1.05)
mid_awake (scale of 1-5), mean (SD)	3.2 (1.31)
sleep_satisfy (scale of 1-5), mean (SD)	2.85 (1.02)
alcohol_amount (cups), mean (SD)	0.3 (0.63)
caffeine_amount (cups), mean (SD)	1.53 (1.46)
worktime_today_duration (h), mean (SD)	5.62 (4.19)
worktime_today_duration (1: midnight-8 AM; h), mean (SD)	1.52 (2.85)
worktime_today_duration (2: 8 AM-4 PM; h), mean (SD)	2.15 (3.25)
worktime_today_duration (3: 4 PM-midnight; h), mean (SD)	1.96 (3.08)
extrawork_activities (n=1865), n (%)	924 (49.54)
sleep_prev_24^d^ (n=2092), n (%)	2088 (99.81)
wake_natural (n=1890), n (%)	793 (41.96)
wake_alarm (n=1890), n (%)	882 (46.67)
wake_other (n=1890), n (%)	198 (10.48)

^a^hrmean: average daily heart rate.

^b^hrstd: SD of daily heart rate.

^c^hrentropy: entropy of daily heart rate.

^d^sleep_prev_24: binary indicator of sleep in the previous 24 hours.

**Table 3:** Mean and SD of participants’ daily well-being scores. Morn indicates morning and eve indicates evening.

Well-being metric	Values, mean (SD)
sleepiness_morn (scale of 1-9)	5.49 (1.99)
sleepiness_now_eve (scale of 1-9)	5.7 (1.78)
sleepiness_daytime (scale of 1-5)	2.59 (1.23)
stress (scale of 1-5)	2.93 (1.1)
tiredness (scale of 1-5)	2.51 (1.01)
alertness_morn (scale of 1-100)	41 (23.02)
happiness_morn (scale of 1-100)	52.99 (17.34)
energy_morn (scale of 1-100)	44.02 (21.27)
health_morn (scale of 1-100)	51.71 (18.12)
calmness_morn (scale of 1-100)	55.97 (18.1)
alertness_eve (scale of 1-100)	41.08 (21.1)
happiness_eve (scale of 1-100)	54.27 (18.18)
energy_eve (scale of 1-100)	45.88 (21.08)
health_eve (scale of 1-100)	52.4 (18.19)
calmness_eve (scale of 1-100)	56.85 (19)

### Advice Statistics

**Figure 2:**
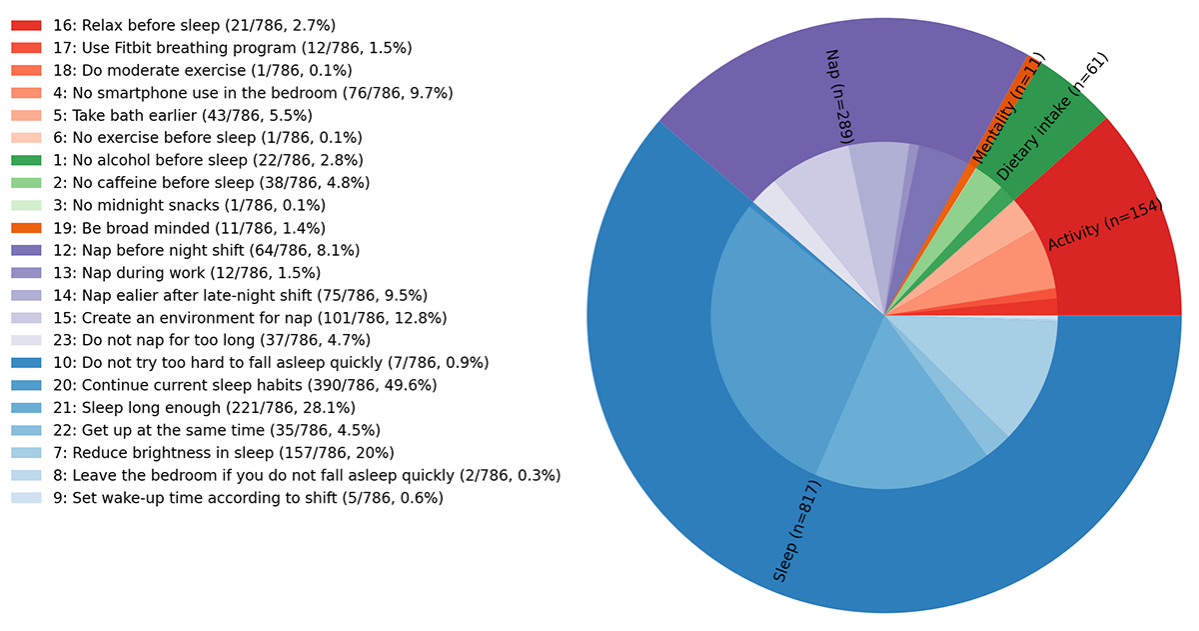
The pie chart shows the selection frequency of each sleep advice message. All 23 message options are divided into 6 categories: dietary intake (messages 1, 2, and 3), activity (messages 4, 5, 6, 16, 17, and 18), sleep (messages 7, 8, 9, 10, 20, 21, and 22), shift (message 11), nap (messages 12, 13, 14, 15, and 23), and mentality (message 19). Message 11 is excluded from the chart because of zero frequency.

Figure 2 shows the frequency of advice provided as well as the message categories in a pie chart. As physicians often selected multiple messages for each day, 1332 advice messages were selected in 786 advice pieces. The outer ring of the pie chart indicates the proportions of each category with respect to the 1332 total occurrences of all message options, and the numbers in parentheses after each category name correspond to the frequency of the category. The inner ring shows the proportion of each message with respect to the total occurrences. The legend lists the corresponding color of each message, and each parenthesis includes the proportion of all advice pieces containing that message, which is computed by dividing the frequency of the message by 786 total advice counts. Messages in the sleep and nap categories were selected more frequently than messages from other categories. Messages in the sleep category were selected the greatest number of times (817/1332, 61.34%), and messages in the nap category were selected 21.7% (289/1332) of the time. Moreover, only a few messages were frequently selected by physicians. For example, message 20 (*continue current sleep habits*) was selected in 49.6% (390/786) of advice pieces, and message 21 (*try to make enough time for sleep*) was selected in 28.1% (221/786) of advice pieces. On the other hand, 70% (16/23) of the messages were selected in <50 advice pieces (50/786, 6.4%), and 7 messages were selected <10 times (10/786, 1.3%).

**Figure 3:**
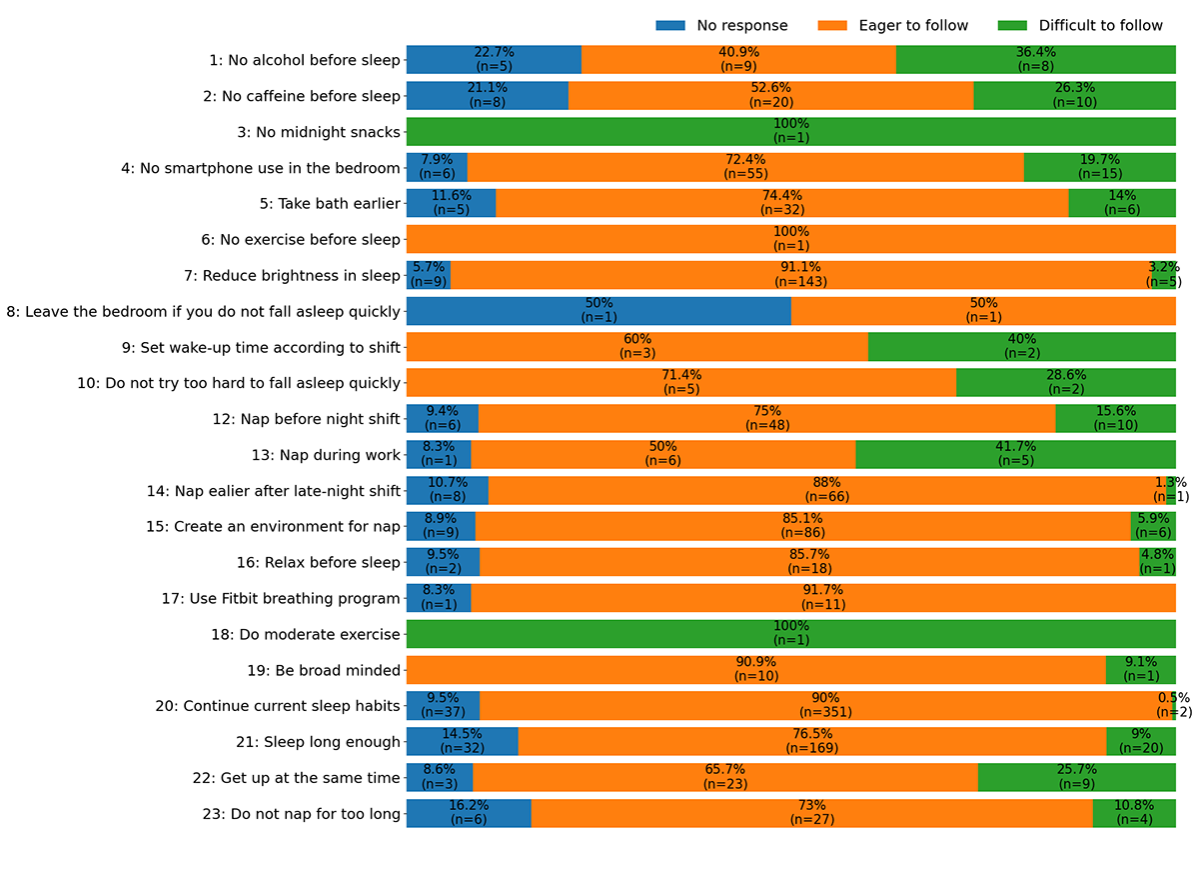
Frequency and proportion of message responses in a stacked bar plot. Each bar includes the frequency and proportion of participants’ responses (no response, eager to follow, and difficult to follow) to advice pieces containing each message. Although multiple messages might be selected for 1 advice piece, participants responded to the advice but not to individual messages.

Figure 3 summarizes the frequency and proportion of responses to advice pieces containing each message. Sometimes, participants did not respond to the advice. Although multiple messages might be selected for 1 advice piece, participants responded to the advice but not to individual messages. Participants showed different preferences for messages. For example, participants expressed difficulty following message 20 (*continue current sleep habits*) only 0.5% (2/390) of the time, whereas they experienced difficulty following message 4 (*refrain from using a smartphone in the bedroom*) 20% (15/76) of the time and following message 2 (*stop consuming caffeine 3 hours before lights-out*) 26% (10/38) of the time. The discrepancies might be explained by the message design. Message 20 suggests that participants continue their current sleep habits, which participants might feel is easier to follow as it would not change their current lifestyle greatly. On the other hand, message 2 and message 4 address caffeine consumption and phone use, which might be challenging to follow as it is suggested that participants change some essential aspects of their routine.

Table S3 in Multimedia Appendix 1 summarizes the distributions of positive and negative advice labels for both the training and test data under user-dependent and user-independent settings. In addition, it includes the ratios of negative-to-positive sample counts. Class imbalances were evident for all messages except message 20 (*continue current sleep habits*). In both data split settings, messages 21 (*try to make enough time for sleep*) and 7 (*darken the bedroom when you go to bed*) showed moderate imbalances, with negative-to-positive ratios of <5. For the remaining 4 messages, class imbalances were more pronounced, with negative-to-positive ratios mostly ranging from 5 to 10. Furthermore, we observed substantial discrepancies in class imbalance between the user-independent training and test sets of messages 12 (*take a nap for approximately 90 minutes before the night shift*), 14 (*take a nap earlier after a late-night shift to refresh*), and 15 (*create an environment for taking a nap*). While negative-to-positive ratios remained <10 in the training sets, the ratios exceeded 15 in the test sets, indicating distinct preferences among physicians when providing sleep advice to different shift workers.

### Advice Prediction Results

Following the approach described in the *Methods* section, we obtained the AUPRC of 9 different configurations of classifiers and data-balancing methods on the test set under dependent and independent settings. Table 4 summarizes the performance of the proposed models, where each model configuration was tuned and trained 10 times with different random seeds, as well as results from 10 random-guessing trials. The model performance was compared with random guessing using a 1-sided Welch *t* test.

As shown in Table 4, under the user-dependent setting, RF performed the best for predicting 6 advice messages. The only exception was advice message 7 (*darken the bedroom when you go to bed*), where CatBoost achieved the highest AUPRC. However, RF’s AUPRC was only 0.02 lower than that of CatBoost. Under the user-independent setting, RF remained competitive, achieving the best performance for predicting 3 advice messages. Its performance on other messages was also close to that of the top performers. LGBM yielded the highest prediction AUPRC for messages 12 (*take a nap for approximately 90 minutes before the night shift*) and 14 (*take a nap earlier after a late-night shift to refresh*). CatBoost was the top performer for messages 7 (*darken the bedroom when you go to bed*) and 21 (*try to make enough time for sleep*). These results highlight the effectiveness of RF in mitigating overfitting when working with limited data. While models trained on balanced data achieved the best performance on 4 advice messages in both dependent and independent settings, data-balancing techniques overall did not result in a substantial improvement in AUPRC and even degraded the performance for some advice labels. Statistical tests revealed that the best prediction models for each advice message and data split setting performed significantly better than random guessing, with *P*<.001 for 13 models and *P*=.003 for the remaining one.

**Table 4:** Advice prediction area under the precision-recall curve (AUPRC) of the proposed models (random forest [RF], light gradient-boosting machine [LGBM], and CatBoost) and random guessing baseline obtained under different data-balancing approaches (none, random oversampling, and synthetic minority oversampling technique [SMOTE]) and dataset split settings (user dependent and user independent). A 1-sided Welch *t* test was used to compare the models to random guessing.

Setting, message ID, and balancing method			RF (AUPRC)		LGBM (AUPRC)			CatBoost (AUPRC)			Random guessing (AUPRC), mean (SD)	
			Values, mean (SD)	*P* value	Values, mean (SD)	*P* value	Values, mean (SD)		*P* value			
**Dependent**												
	**4**											0.12 (0.02)
		None	*0.33 (0.07)* ^a^	*<.001*	0.28 (0.06)	<.001	0.24 (0.04)		<.001			
		Random oversampling	0.25 (0.05)	<.001	0.21 (0.04)	<.001	0.20 (0.03)		<.001			
		SMOTE	0.24 (0.03)	<.001	0.18 (0.02)	<.001	0.18 (0.02)		<.001			
	**7**											0.17 (0.02)
		None	0.56 (0.04)	<.001	0.54 (0.01)	<.001	0.57 (0.02)		<.001			
		Random oversampling	0.54 (0.03)	<.001	0.52 (0.03)	<.001	0.54 (0.03)		<.001			
		SMOTE	0.52 (0.03)	<.001	0.56 (0.02)	<.001	*0.58 (0.02)*		*<.001*			
	**12**											0.12 (0.02)
		None	*0.32 (0.03)*	*<.001*	0.23 (0.03)	<.001	0.27 (0.06)		<.001			
		Random oversampling	0.31 (0.04)	<.001	0.21 (0.04)	<.001	0.26 (0.07)		<.001			
		SMOTE	0.19 (0.03)	<.001	0.17 (0.01)	<.001	0.22 (0.05)		<.001			
	**14**											0.10 (0.02)
		None	0.26 (0.05)	<.001	0.24 (0.04)	<.001	0.23 (0.05)		<.001			
		Random oversampling	0.25 (0.04)	<.001	0.22 (0.04)	<.001	0.23 (0.05)		<.001			
		SMOTE	*0.29 (0.05)*	*<.001*	0.22 (0.05)	<.001	0.22 (0.04)		<.001			
	**15**											0.19 (0.03)
		None	*0.31 (0.03)*	*<.001*	0.26 (0.05)	.001	0.25 (0.05)		.007			
		Random oversampling	0.28 (0.02)	<.001	0.27 (0.05)	<.001	0.22 (0.04)		.06			
		SMOTE	0.25 (0.01)	<.001	0.24 (0.02)	<.001	0.24 (0.02)		.002			
	**20**											0.57 (0.02)
		None	0.80 (0.02)	<.001	0.76 (0.02)	<.001	0.79 (0.02)		<.001			
		Random oversampling	0.80 (0.01)	<.001	0.77 (0.02)	<.001	0.80 (0.02)		<.001			
		SMOTE	*0.81 (0.02)*	*<.001*	0.77 (0.02)	<.001	0.79 (0.01)		<.001			
	**21**											0.23 (0.02)
		None	0.38 (0.03)	<.001	0.38 (0.03)	<.001	0.41 (0.01)		<.001			
		Random oversampling	0.38 (0.03)	<.001	0.36 (0.02)	<.001	0.40 (0.03)		<.001			
		SMOTE	*0.44 (0.04)*	*<.001*	0.40 (0.04)	<.001	0.44 (0.06)		<.001			
**Independent**												
	**4**											0.11 (0.05)
		None	*0.25 (0.03)*	*<.001*	0.17 (0.04)	.005	0.20 (0.03)		<.001			
		Random oversampling	0.19 (0.04)	.001	0.16 (0.04)	.01	0.15 (0.03)		.02			
		SMOTE	0.18 (0.06)	.007	0.12 (0.04)	.25	0.11 (0.02)		.58			
	**7**											0.16 (0.02)
		None	0.67 (0.05)	<.001	0.61 (0.03)	<.001	0.66 (0.03)		<.001			
		Random oversampling	0.60 (0.08)	<.001	0.58 (0.06)	<.001	0.62 (0.04)		<.001			
		SMOTE	0.66 (0.06)	<.001	0.64 (0.04)	<.001	*0.70 (0.04)*		*<.001*			
	**12**											0.03 (0.01)
		None	0.07 (0.01)	<.001	*0.09 (0.01)*	*<.001*	0.06 (0.01)		<.001			
		Random oversampling	0.06 (0.01)	<.001	0.08 (0.02)	<.001	0.07 (0.02)		<.001			
		SMOTE	0.07 (0.01)	<.001	0.06 (0.01)	<.001	0.06 (0.01)		<.001			
	**14**											0.06 (0.02)
		None	0.09 (0.01)	.001	0.07 (0.01)	.18	0.10 (0.02)		<.001			
		Random oversampling	0.10 (0.01)	<.001	0.07 (0.02)	.11	0.09 (0.01)		.001			
		SMOTE	0.09 (0.02)	.002	*0.11 (0.04)*	*.003*	0.09 (0.03)		.004			
	**15**											0.06 (0.01)
		None	0.11 (0.02)	<.001	0.09 (0.03)	.003	0.09 (0.02)		.002			
		Random oversampling	*0.11 (0.03)*	*<.001*	0.10 (0.02)	<.001	0.10 (0.02)		<.001			
		SMOTE	0.08 (0.03)	.04	0.07 (0.01)	.07	0.08 (0.02)		.03			
	**20**											0.53 (0.02)
		None	0.64 (0.01)	<.001	0.64 (0.01)	<.001	0.62 (0.01)		<.001			
		Random oversampling	0.64 (0.01)	<.001	0.64 (0.02)	<.001	0.63 (0.01)		<.001			
		SMOTE	*0.64 (0.01)*	*<.001*	0.64 (0.02)	<.001	0.63 (0.01)		<.001			
	**21**											0.24 (0.03)
		None	0.36 (0.01)	<.001	0.38 (0.02)	<.001	0.34 (0.02)		<.001			
		Random oversampling	0.35 (0.02)	<.001	0.36 (0.03)	<.001	0.35 (0.03)		<.001			
		SMOTE	0.38 (0.02)	<.001	0.36 (0.03)	<.001	*0.39 (0.02)*		*<.001*			

^a^The best-performing configuration for each sleep advice message is italicized.

### Important Features

We extracted permutation and SHAP feature importance for the best model configuration when predicting each advice message under both user-dependent and user-independent settings. Table 5 shows the features that ranked among the top 5 most important using both permutation importance and SHAP, with average AUPRC decrease and mean absolute SHAP values of at least 0.01. All the features represented the average and SD of daily Fitbit and survey features across the previous 4 days for each date on which physicians provided advice. As expected, several important features closely matched their corresponding advice messages under both data split settings. The average amount of phone use before sleep was a key factor in predicting message 4 (*refrain from using a smartphone in the bedroom*). Similarly, the average brightness of the sleep environment was the most significant feature for predicting message 7 (*darken the bedroom when you go to bed*). The model examined average sleep duration for predictions of message 21 (*try to make enough time for sleep*). When predicting message 12 (*take a nap for approximately 90 minutes before the night shift*), the SD of work hours between 4 PM and midnight on the current day across the previous 4 days was an important factor. This suggests a potential relationship between the frequency of night shifts and the triggering of this sleep advice. In addition, the response to the previous selection of message 20 (*continue current sleep habits*) contributed to the prediction of messages 7, 20, and 21. This aligns with physicians’ insights that they often considered participants’ responses to previous advice when providing sleep recommendations. These findings demonstrate the models’ ability to capture meaningful patterns that resonate with physicians’ decision-making processes.

**Table 5:** Important features identified using permutation importance and Shapley Additive Explanations (SHAP) for the best model configuration when predicting each advice message under both user-dependent and user-independent settings. Features are listed if they ranked among the top 5 most important using both permutation importance and SHAP, with average area under the precision-recall curve (AUPRC) decrease and mean absolute SHAP values of at least 0.01.

Setting, message ID, and feature				AUPRC decrease, mean (SD)		Absolute SHAP value, mean (SD)
**Dependent**						
	**4**					
		Average amount of phone use before sleep	0.19 (0.06)		0.05 (0.01)	
	**7**					
		Average brightness of sleep environment	0.33 (0.05)		1.14 (0.25)	
		Response to previous selection of message 20	0.04 (0.03)		0.29 (0.06)	
		SD of brightness of sleep environment	0.03 (0.02)		0.18 (0.06)	
		SD of the binary indicator of activities other than work outside the homes	0.02 (0.01)		0.09 (0.04)	
	**12**					
		SD of work hours on the current day for 4 PM-midnight	0.05 (0.07)		0.02 (0.01)	
	**14**					
		SD of daily caffeine intake	0.03 (0.02)		0.02 (0.01)	
	**20**					
		Response to previous selection of message 20	0.12 (0.03)		0.10 (0.04)	
		Average sleep satisfaction	0.01 (0.01)		0.02 (0.01)	
		Average fatigue recovery level	0.01 (0.01)		0.03 (0.01)	
	**21**					
		Response to previous selection of message 20	0.11 (0.05)		0.07 (0.03)	
		Average sleep duration	0.03 (0.02)		0.02 (0.005)	
		SD of work hours on the current day for midnight-8 AM	0.03 (0.03)		0.01 (0.01)	
**Independent**						
	**4**					
		Average amount of phone use before sleep	0.13 (0.04)		0.09 (0.02)	
	**7**					
		Average brightness of sleep environment	0.47 (0.05)		1.35 (0.39)	
		Response to previous selection of message 20	0.03 (0.02)		0.18 (0.06)	
	**12**					
		SD of work hours on the current day for 4 PM-midnight	0.03 (0.02)		0.28 (0.10)	
	**14**					
		Response to previous selection of message 20	0.03 (0.04)		0.25 (0.10)	
	**15**					
		Average sleep satisfaction	0.02 (0.03)		0.05 (0.04)	
		Average fatigue recovery level	0.02 (0.02)		0.02 (0.01)	
	**20**					
		Response to previous selection of message 20	0.11 (0.02)		0.16 (0.03)	
		Average sleep satisfaction	0.02 (0.01)		0.03 (0.01)	
	**21**					
		Response to previous selection of message 20	0.09 (0.04)		0.45 (0.07)	
		Average sleep duration	0.02 (0.03)		0.17 (0.08)	

## Discussion

### Principal Findings

In this study, we used daily surveys and Fitbit to obtain physiological and behavioral data of ICU shift workers. Physicians reviewed the data to provide sleep advice from a list of 23 messages to the workers. We extracted features from the collected data and (1) conducted analyses to characterize participants’ behaviors and advice and (2) constructed and evaluated 3 models (RF, LGBM, and CatBoost) and 3 data-balancing approaches (no balancing, random oversampling, and SMOTE) to predict sleep advice selections for the 7 most frequent messages sent by the physicians. The advice prediction models achieved higher AUPRC than the random guessing baseline for predicting all the 7 advice messages under both user-dependent and user-independent settings. The performance differences were statistically significant, with *P*<.001 for 13 tests and *P*=.003 for 1 test. Feature importance analysis revealed several important features that closely matched their corresponding advice messages sent to participants. For instance, for message 7 (*darken the bedroom when you go to bed*), the models primarily examined the average brightness of the sleep environment to make predictions. Similarly, the average amount of phone use before sleep was a key factor in predicting message 4 (*refrain from using a smartphone in the bedroom*). The analysis also supported physicians’ insights that they often considered participants’ responses to previous advice when providing sleep recommendations.

### Participant Characteristics

Hierarchical clustering revealed different work shifts and behavior patterns among clusters and subclusters, and we investigated their relationships with their well-being scores. First, work shifts on the current day contributed to low well-being scores for clusters 3 and 5, and several well-being metrics were further distinguished by their different shift patterns. For example, cluster 3 had a much shorter average sleep duration of approximately 220 minutes and the lowest sleep quality. This was potentially caused by day shifts the day before and midnight shifts on the current day for almost all samples. In this cluster, participants finished their work at approximately 4 PM the day before; they then started the next shift period at approximately midnight and finished at approximately 8 AM on the following day. After work, they filled out the morning surveys and reported their well-being, sleep duration, and sleep quality. Low scores on these metrics reflected their tiredness shortly after work and lack of rest between work shifts.

On the other hand, most samples in cluster 5 did not have shifts the day before but had day shifts on the current day. As a result, participants could have more time for sleep, reflected by a longer average sleep duration of 400 minutes, better sleep quality, and higher well-being scores in the morning compared with samples in cluster 3. Such discrepancies in shift patterns and well-being metrics could even be observed between subclusters *d* and *k* of the same larger cluster 2. Although most samples of both subclusters had midnight shifts the day before, subcluster *d* majorly did not have shifts on the current day and achieved an average level of well-being scores. The profile of subcluster *k* was more similar to that of cluster 3, with midnight shifts on the current day and much worse well-being and sleep quality than in subcluster *d*, which is also supported by the t-SNE plot (right plot in Figure S1 in Multimedia Appendix 1), where subcluster *k* is closer to subcluster *h* (equivalence of cluster 3) than to subcluster *d*. Furthermore, most of cluster 1 did not have a work shift on the current day, which led to its high well-being scores as 1 day off probably helped participants relax. Nevertheless, shifts on the current day were not always negatively correlated with well-being. Specifically, cluster 0 had above-average well-being scores although most samples had work shifts on the current day.

Furthermore, several subclusters were characterized by some distinct features of their profiles. For example, subclusters *i* and *l* of cluster 5 had similar work patterns, but they differed significantly in average daily step count, where subcluster *i* had the highest count of >10,000 among all subclusters, whereas subcluster *l* had the lowest count of <2000. Although multiple clusters and subclusters contained samples with overtime shifts of >8 hours, subcluster *e* was the only one with >9 hours of average work duration for 3 consecutive days (10.6 hours the day before the day before, 9.4 hours the day before, and 11.5 hours on the current day). The long work duration might also contribute to other characteristics of this subcluster, including the shortest time to fall asleep and the highest daily caffeine consumption. Despite the existence of some outliers, the clusters and subclusters generally grouped well, as shown in Figure S1 in Multimedia Appendix 1, and their profiles summarized from average feature values provided many insights into participants’ daily behaviors, shifts, and well-being and their interrelationships.

### Advice Predictions

While we evaluated the advice prediction models using the AUPRC computed from predicted class probabilities, it is also important to understand the model performance in terms of actual predicted classes. Given the class imbalances, adjusting the decision thresholds was necessary to optimize prediction performance. Specifically, for each classifier initialized with its optimal hyperparameters, we performed 5-fold stratified cross-validation on the training set to determine the optimal threshold that maximized its *F*_1_-score. The *F*_1_-score was chosen as the performance metric to obtain a balance between missed advice predictions and false alarms. The optimal threshold was then applied to convert prediction probabilities into class labels on the test set. As we repeated the tuning and training process 10 times with different random seeds for each model configuration, we created an ensemble of these classifiers to mitigate potential overfitting. Final predictions were determined through majority voting across the ensemble. To resolve ties during voting, we used only 9 of the 10 classifiers generated for each model configuration. We report the sensitivity, specificity, precision, and *F*_1_-scores of the ensembles from the RF, LGBM, and CatBoost classifiers with different data-balancing approaches in Tables S4, S5, and S6 in Multimedia Appendix 1, respectively.

Table 6 presents the performance of the model configurations with the highest *F*_1_-score for predicting each sleep advice message under user-dependent and user-independent settings. Baseline *F*_1_-scores, calculated by always predicting the positive class, are also provided for comparison. All models outperformed the baseline. Under the user-dependent setting, the models achieved sensitivity and specificity within the range of 0.5 to 0.9. Sensitivity tended to decrease for less frequent advice messages, whereas specificity showed the opposite trend. Under the user-independent setting, the models consistently demonstrated decent sensitivity, exceeding 0.7 across all advice messages, whereas specificity varied between 0.4 and 0.9. The higher sensitivity observed in this setting may suggest that the models were more effective at identifying universal decision rules shared across participants than at accounting for individual behavioral variability.

**Table 6:** Advice prediction sensitivity, specificity, precision, and F1-score of the best-performing ensemble classifier for each advice message under both user-dependent and user-independent settings. Ensemble classifiers were constructed through majority voting across 9 random forest (RF), light gradient-boosting machine (LGBM), or CatBoost classifiers trained using different random seeds. The decision threshold for each classifier was optimized to maximize the F1-score. The baseline F1-score from always predicting the positive class is also listed.

Setting and message ID		Model	Balancing method	Sensitivity	Specificity	Precision	*F*_1_-score	Baseline *F*_1_-score (always positive)
**Dependent**								
	4	LGBM	None	0.56	0.86	0.34	0.42	0.20
	7	CatBoost	Random oversampling	0.69	0.88	0.52	0.59	0.28
	12	RF	None	0.50	0.83	0.27	0.35	0.19
	14	RF	Random oversampling	0.58	0.81	0.27	0.37	0.19
	15	RF	Random oversampling	0.55	0.72	0.29	0.38	0.30
	20	RF	SMOTE^a^	0.84	0.50	0.67	0.75	0.71
	21	CatBoost	None	0.81	0.58	0.34	0.48	0.35
**Independent**								
	4	RF	None	0.95	0.54	0.17	0.29	0.17
	7	CatBoost	SMOTE	0.73	0.93	0.63	0.68	0.25
	12	LGBM	Random oversampling	1.00	0.60	0.07	0.14	0.06
	14	CatBoost	None	0.91	0.68	0.12	0.22	0.09
	15	RF	SMOTE	0.77	0.48	0.08	0.15	0.11
	20	RF	SMOTE	0.82	0.46	0.61	0.70	0.67
	21	RF	Random oversampling	0.81	0.44	0.31	0.45	0.38

^a^SMOTE: synthetic minority oversampling technique.

### Single-Label Versus Multilabel Classification

As physicians could select multiple messages when providing sleep advice, it is feasible to formulate sleep advice prediction as a multilabel classification task, which outputs predictions for all message selections simultaneously. Compared to the current approach of training separate single-label classifiers for each message, multilabel classification has the advantage of leveraging information about message coselections during training, potentially improving prediction performance. However, this approach presents several challenges. First, some models do not inherently support multilabel classification and, instead, combine separate single-label classifiers for multilabel outputs. This strategy fails to capture and use the coselection information effectively. Second, the multilabel setting requires careful handling of data imbalances both within individual labels and across their co-occurrence patterns. Finally, multilabel classification complicates feature importance analysis, which aims to identify key contributors for predicting individual message selections. In fact, we trained a user-dependent multilabel RF classifier without any data balancing and did not observe substantial performance improvement compared to the single-label counterparts.

### Comparison With Prior Work

Our system provided sleep and behavior recommendations for shift workers with poor sleep quality. Although our system did not follow the CBTI protocol, we borrowed elements from it, such as sleep hygiene advice and relaxation. We designed sleep recommendations specifically targeted for shift workers, including strategic napping and clockwise shift rotation. Our system offered several advantages over existing CBTI and sleep RSs. First, while most mobile and web-based CBTI systems require users to review sleep-related learning modules, our system reduced users’ time commitment by sending them simple pieces of advice to follow, which could be beneficial as shift workers often have a poor work-life balance [53]. Consequently, our study yielded a low dropout rate of 5% (3/64), as opposed to the rates of other CBTI systems that range from 4% to 61% [15,16,18-23,26,29,54].

Moreover, our system received the most support from sleep specialists compared to existing sleep RSs, which only have limited or even no clinician support. For example, clinicians only provided recommendation templates for further customization by the automated SleepCoacher system [30]. The cohort-based sleep recommender by Daskalova et al [31] relied on profiles of similar users as the only recommendation source. The system developed by Pandey et al [32] constructed its recommendations based on discovered relationships between the user’s lifestyle and sleep. The lack of clinician support could impair the credibility of the provided sleep recommendations among users. For instance, users of SleepCoacher suggested adding justifications for the advice [30], and several users of the cohort-based sleep recommender by Daskalova et al [31] thought that the recommendations were implausible and even untrustworthy as they contradicted the users’ own beliefs. However, our system only delivered clinician-reviewed recommendations, which greatly enhanced their trustworthiness.

Furthermore, only our study has evaluated the accuracy of an algorithm for automatic sleep advice provision. Many existing CBTI systems have been able to provide sleep restriction and sleep hygiene recommendations automatically, but no analysis is available of how close these generated recommendations are to actual clinicians’ advice. Similarly, sleep RSs have not compared their recommendations with professionals’ suggestions. Specifically, clinicians prepared all the recommendations in the preliminary study of SleepCoacher, and the provisions were automated by a correlation-based algorithm in the final study, but the study did not evaluate the accuracy of the algorithm for substituting clinicians [30]. Despite the effectiveness of these systems, an analysis of algorithm accuracy could further justify provided recommendations and resolve users’ doubts about their credibility. On the other hand, although our system is not automated at its current stage, we developed an algorithm and assessed its accuracy for automatic advice provision, and it showed decent performance and great potential in providing clinical-level sleep recommendations.

### Limitations and Future Work

#### False Predictions

To investigate the possible causes of false-positive and false-negative predictions, we examined the relationship between feature values and advice predictions. Specifically, we chose the feature *brightness_sleep* (brightness level of the sleep environment) and 1 repetition of the dependent advice prediction model for message 7 (*darken the bedroom when you go to bed*). We selected this combination because the feature had a dominant effect on predictions of the message label, as shown in the *Important Features* section, and could demonstrate the limitations of the models more clearly with the existence of an explicit decision rule.

For each participant, we plotted the trend of the feature across all days of their enrollment and marked each predicted and the actual selection of the message separately. Figure S2 in Multimedia Appendix 1 includes such plots for 6 participants to illustrate incorrect predictions and limitations of the advice prediction models. First, the models did not have memories about previous message selections and might produce false positives as duplicate responses to previous feature values. For example, the model made 3 positive predictions on days 29, 31, and 33 in response to a peak of brightness on day 29 for participant 1102, yet only the prediction on day 29 was an actual positive. Similarly, 2 positive predictions were made for participant 1154 on days 26 and 29 for a brightness surge on day 26, but the latter was a false-positive. Shortening the feature averaging window before each message selection might eliminate some of these false positives, but it would increase the risk of producing false negatives when a day with a favorable value for message selection is excluded from the window.

Another scenario of false positives occurred for participants 1315 and 1181. Both participants received 4 false positives although their brightness values only fluctuated within a small range of approximately 30 (scale of 1-100). A closer look at individual decision trees of the trained RF showed that some trees considered SDs of brightness levels but most trees made predictions by comparing average brightness with a threshold. Thus, false-positive predictions were made because average brightness exceeded the thresholds of most decision trees despite the lack of brightness peaks. However, it could be tricky to make correct predictions under such a scenario. For example, physicians selected the message twice on days 9 and 16 for participant 1315 although brightness remained stable. A plausible explanation could be that physicians initially suggested that the participant lower the brightness because it was constantly above the threshold, but later, they stopped as neither lower levels nor high peaks of brightness were observed after the suggestions. Nevertheless, it would be very difficult for the model to capture such change.

Unlike false positives, most false negatives occurred only under 1 scenario: when brightness peaks were not high enough and adjacent days had very low feature values, which caused averages to fall below the threshold, for example, on day 33 for participant 1164 and on days 29, 31, and 33 for participant 1167. Measuring the relative height of a peak (difference between maximum and minimum values of the feature averaging window) instead of averaging might be helpful to eliminate these false negatives, but it may produce other false negatives when feature values remain high and stable. From illustrative plots of feature values and advice predictions, we demonstrated several causes of false-positive and false-negative predictions, but further investigations and experiments were necessary to properly improve the advice prediction models as some false positives might benefit participants and strategies to eliminate certain false predictions might introduce others.

#### System Usability

Although our developed algorithm achieved a decent performance in predicting sleep advice under both dependent and independent settings, the algorithm is limited to the 7 most popular advice messages among 23 choices due to infrequent occurrences of the remaining options. Therefore, further studies are necessary to gather enough data to enable predictions for less frequent advice labels.

We gathered feedback on the sleep advice models from clinicians (n=2) through questionnaires. They commented that they decided their suggestions mainly based on morning survey, work shift, and wearable data. After they selected the suggestions, they referred to the output of the models and made the final decisions.

Future studies can compare the intervention effects of a clinician-only input system, an algorithm-assisted system, and a fully automated system to demonstrate the usefulness of the machine learning algorithm. It will also be beneficial to validate the generalizability of our advice prediction models with more diverse participant populations.

Given the effectiveness of existing sleep RSs with limited clinical support, we believe that our automated system has the advantage of being built on the knowledge of sleep specialists even if our algorithm still requires improvement to further boost performance. We chose to evaluate our models in user-dependent and user-independent settings instead of using leave-one-out cross-validation because the advice frequencies were extremely imbalanced across participants. However, such a scheme will fit real-world scenarios the best when models use all available data from current users to predict sleep advice for a new user. It is also worth experimenting with personalization beyond leave-one-out cross-validation by continuously incorporating data accumulated from new users as they progress to better adapt models to specific user characteristics.

### Conclusions

We developed a web- and app-based system to provide individualized sleep and behavior advice with CBTI elements for shift workers. We collected data from shift workers (N=61) in the ICU at 2 hospitals in Japan for 5 weeks, which included Fitbit, survey, and sleep advice data. We used hierarchical clustering to characterize shift workers’ physiological and behavioral features and identified clusters with distinct patterns. For example, days with low sleep duration and the lowest sleep quality were associated with a day shift on the day before and a midnight shift on the current day. Furthermore, we constructed and evaluated 3 machine learning models (RF, LGBM, and CatBoost) and 3 data-balancing approaches (no balancing, random oversampling, and SMOTE) to predict sleep advice selections for the 7 most frequent advice messages sent by the physicians. Our advice prediction models achieved higher AUPRC and *F*_1_-scores compared to the baseline. The analysis of the feature importance of our models found several important features that closely matched their corresponding advice messages sent to participants. Although our current system requires physician input, an accurate machine learning algorithm would be promising for automation without hurting the trustworthiness of the selected recommendations. Despite a decent performance, the algorithms developed in this study are limited to the 7 most popular advice messages among 23 choices due to infrequent occurrences of the remaining advice options. Therefore, further studies are necessary to gather enough data to enable predictions for less frequent advice labels.

## Appendix

Multimedia Appendix 1 Additional tables and figures for participant characteristic clustering and advice prediction models.

## Abbreviations

AUPRC: area under the precision-recall curve
CBTI: cognitive behavioral therapy for insomnia
ICU: intensive care unit
LGBM: light gradient-boosting machine
RF: random forest
RS: recommender system
SHAP: Shapley additive explanations
SMOTE: synthetic minority oversampling technique
SRI: sleep regularity index
SWSD: shift work sleep disorder
t-SNE: t-distributed stochastic neighbor embedding

## Data Availability

The datasets generated or analyzed during this study are available from the corresponding author on reasonable request.

## References

[1] Alterman T, Luckhaupt SE, Dahlhamer JM, Ward BW, Calvert GM (2013). Prevalence rates of work organization characteristics among workers in the U.S.: data from the 2010 National Health Interview Survey. Am J Ind Med.

[2] Wickwire EM, Geiger-Brown J, Scharf SM, Drake CL (2017). Shift work and shift work sleep disorder: clinical and organizational perspectives. Chest.

[3] Haile KK, Asnakew S, Waja T, Kerbih HB (2019). Shift work sleep disorders and associated factors among nurses at federal government hospitals in Ethiopia: a cross-sectional study. BMJ Open.

[4] Flo E, Pallesen S, Magerøy N, Moen BE, Grønli J, Hilde Nordhus I, Bjorvatn B (2012). Shift work disorder in nurses--assessment, prevalence and related health problems. PLoS One.

[5] Di Milia L, Waage S, Pallesen S, Bjorvatn B (2013). Shift work disorder in a random population sample--prevalence and comorbidities. PLoS One.

[6] Cheng WJ, Cheng Y (2017). Night shift and rotating shift in association with sleep problems, burnout and minor mental disorder in male and female employees. Occup Environ Med.

[7] Khan WA, Jackson ML, Kennedy GA, Conduit R (2021). A field investigation of the relationship between rotating shifts, sleep, mental health and physical activity of Australian paramedics. Sci Rep.

[8] Saleh AM, Awadalla NJ, El-masri YM, Sleem WF (2014). Impacts of nurses’ circadian rhythm sleep disorders, fatigue, and depression on medication administration errors. Egypt J Chest Dis Tuberc.

[9] Blytt KM, Bjorvatn B, Moen BE, Pallesen S, Harris A, Waage S (2022). The association between shift work disorder and turnover intention among nurses. BMC Nurs.

[10] Wright KP, Bogan RK, Wyatt JK (2013). Shift work and the assessment and management of shift work disorder (SWD). Sleep Med Rev.

[11] Qaseem A, Kansagara D, Forciea MA, Cooke M, Denberg TD, Clinical Guidelines Committee of the American College of Physicians (2016). Management of chronic insomnia disorder in adults: a clinical practice guideline from the American College of Physicians. Ann Intern Med.

[12] Williams J, Roth A, Vatthauer K, McCrae CS (2013). Cognitive behavioral treatment of insomnia. Chest.

[13] Järnefelt H, Lagerstedt R, Kajaste S, Sallinen M, Savolainen A, Hublin C (2012). Cognitive behavioral therapy for shift workers with chronic insomnia. Sleep Med.

[14] Järnefelt H, Sallinen M, Luukkonen R, Kajaste S, Savolainen A, Hublin C (2014). Cognitive behavioral therapy for chronic insomnia in occupational health services: analyses of outcomes up to 24 months post-treatment. Behav Res Ther.

[15] Järnefelt H, Härmä M, Sallinen M, Virkkala J, Paajanen T, Martimo KP, Hublin C (2020). Cognitive behavioural therapy interventions for insomnia among shift workers: RCT in an occupational health setting. Int Arch Occup Environ Health.

[16] Peter L, Reindl R, Zauter S, Hillemacher T, Richter K (2019). Effectiveness of an online CBT-I intervention and a face-to-face treatment for shift work sleep disorder: a comparison of sleep diary data. Int J Environ Res Public Health.

[17] Retzer L, Feil M, Reindl R, Richter K, Lehmann R, Stemmler M, Graessel E (2021). Anonymous online cognitive behavioral therapy for sleep disorders in shift workers-a study protocol for a randomized controlled trial. Trials.

[18] Ritterband LM, Thorndike FP, Gonder-Frederick LA, Magee JC, Bailey ET, Saylor DK, Morin CM (2009). Efficacy of an internet-based behavioral intervention for adults with insomnia. Arch Gen Psychiatry.

[19] Espie CA, Kyle SD, Williams C, Ong JC, Douglas NJ, Hames P, Brown JS (2012). A randomized, placebo-controlled trial of online cognitive behavioral therapy for chronic insomnia disorder delivered via an automated media-rich web application. Sleep.

[20] Batterham PJ, Christensen H, Mackinnon AJ, Gosling JA, Thorndike FP, Ritterband LM, Glozier N, Griffiths KM (2017). Trajectories of change and long-term outcomes in a randomised controlled trial of internet-based insomnia treatment to prevent depression. BJPsych Open.

[21] Hagatun S, Vedaa Ø, Nordgreen T, Smith OR, Pallesen S, Havik OE, Bjorvatn B, Thorndike FP, Ritterband LM, Sivertsen B (2019). The short-term efficacy of an unguided internet-based cognitive-behavioral therapy for insomnia: a randomized controlled trial with a six-month nonrandomized follow-up. Behav Sleep Med.

[22] Cheng P, Luik AI, Fellman-Couture C, Peterson E, Joseph CL, Tallent G, Tran KM, Ahmedani BK, Roehrs T, Roth T, Drake CL (2019). Efficacy of digital CBT for insomnia to reduce depression across demographic groups: a randomized trial. Psychol Med.

[23] Freeman D, Sheaves B, Goodwin GM, Yu LM, Nickless A, Harrison PJ, Emsley R, Luik AI, Foster RG, Wadekar V, Hinds C, Gumley A, Jones R, Lightman S, Jones S, Bentall R, Kinderman P, Rowse G, Brugha T, Blagrove M, Gregory AM, Fleming L, Walklet E, Glazebrook C, Davies EB, Hollis C, Haddock G, John B, Coulson M, Fowler D, Pugh K, Cape J, Moseley P, Brown G, Hughes C, Obonsawin M, Coker S, Watkins E, Schwannauer M, MacMahon K, Siriwardena AN, Espie CA (2017). The effects of improving sleep on mental health (OASIS): a randomised controlled trial with mediation analysis. Lancet Psychiatry.

[24] Beun RJ, Brinkman WP, Fitrianie S, Griffioen-Both F, Horsch C, Lancee J, Spruit S (2016). Improving adherence in automated e-coaching. Proceedings of the 11th International Conference on Persuasive Technology.

[25] Beun RJ, Fitrianie S, Griffioen-Both F, Spruit S, Horsch C, Lancee J, Brinkman WP (2017). Talk and Tools: the best of both worlds in mobile user interfaces for e-coaching. Pers Ubiquit Comput.

[26] Horsch CH, Lancee J, Griffioen-Both F, Spruit S, Fitrianie S, Neerincx MA, Beun RJ, Brinkman W (2017). Mobile phone-delivered cognitive behavioral therapy for insomnia: a randomized waitlist controlled trial. J Med Internet Res.

[27] Kuhn E, Weiss BJ, Taylor KL, Hoffman JE, Ramsey KM, Manber R, Gehrman P, Crowley JJ, Ruzek JI, Trockel M (2016). CBT-I coach: a description and clinician perceptions of a mobile app for cognitive behavioral therapy for insomnia. J Clin Sleep Med.

[28] Koffel E, Kuhn E, Petsoulis N, Erbes CR, Anders S, Hoffman JE, Ruzek JI, Polusny MA (2018). A randomized controlled pilot study of CBT-I coach: feasibility, acceptability, and potential impact of a mobile phone application for patients in cognitive behavioral therapy for insomnia. Health Informatics J.

[29] Kuhn E, Miller KE, Puran D, Wielgosz J, YorkWilliams SL, Owen JE, Jaworski BK, Hallenbeck HW, McCaslin SE, Taylor KL (2022). A pilot randomized controlled trial of the insomnia coach mobile app to assess its feasibility, acceptability, and potential efficacy. Behav Ther.

[30] Daskalova N, Metaxa-Kakavouli D, Tran A, Nugent N, Boergers J, McGeary J, Huang J (2016). SleepCoacher: a personalized automated self-experimentation system for sleep recommendations. Proceedings of the 29th Annual Symposium on User Interface Software and Technology.

[31] Daskalova N, Lee B, Huang J, Ni C, Lundin J (2018). Investigating the effectiveness of cohort-based sleep recommendations. Proc ACM Interact Mob Wearable Ubiquitous Technol.

[32] Pandey V, Upadhyay D, Nag N, Jain R (2020). Personalized user modelling for context-aware lifestyle recommendations to improve sleep. Proceedings of the 5th International Workshop on Health Recommender Systems.

[33] Kay M, Choe EK, Shepherd J, Greenstein B, Watson N, Consolvo S, Kientz JA (2012). Lullaby: a capture and access system for understanding the sleep environment. Proceedings of the 2012 ACM Conference on Ubiquitous Computing.

[34] Bauer J, Consolvo S, Greenstein B, Schooler J, Wu E, Watson N, Kientz J (2012). ShutEye: encouraging awareness of healthy sleep recommendations with a mobile, peripheral display. Proceedings of the 2012 SIGCHI Conference on Human Factors in Computing Systems.

[35] Daskalova N, Yoon J, Wang Y, Araujo C, Beltran G, Nugent N, McGeary J, Williams J, Huang J (2020). SleepBandits: guided flexible self-experiments for sleep. Proceedings of the 2020 CHI Conference on Human Factors in Computing Systems.

[36] Ito-Masui A, Kawamoto E, Sakamoto R, Yu H, Sano A, Motomura E, Tanii H, Sakano S, Esumi R, Imai H, Shimaoka M (2021). Internet-based individualized cognitive behavioral therapy for shift work sleep disorder empowered by well-being prediction: protocol for a pilot study. JMIR Res Protoc.

[37] Ito-Masui A, Sakamoto R, Matsuo E, Kawamoto E, Motomura E, Tanii H, Yu H, Sano A, Imai H, Shimaoka M (2023). Effect of an internet-delivered cognitive behavioral therapy-based sleep improvement app for shift workers at high risk of sleep disorder: single-arm, nonrandomized trial. J Med Internet Res.

[38] Yu H, Itoh A, Sakamoto R, Shimaoka M, Sano A (2020). Forecasting health and wellbeing for shift workers using job-role based deep neural network. Proceedings of the 9th EAI International Conference on Wireless Mobile Communication and Healthcare.

[39] Richman JS, Moorman JR (2000). Physiological time-series analysis using approximate entropy and sample entropy. Am J Physiol Heart Circ Physiol.

[40] Phillips AJ, Clerx WM, O'Brien CS, Sano A, Barger LK, Picard RW, Lockley SW, Klerman EB, Czeisler CA (2017). Irregular sleep/wake patterns are associated with poorer academic performance and delayed circadian and sleep/wake timing. Sci Rep.

[41] Lunsford-Avery JR, Engelhard MM, Navar AM, Kollins SH (2018). Validation of the sleep regularity index in older adults and associations with cardiometabolic risk. Sci Rep.

[42] Murtagh F, Legendre P (2014). Ward’s hierarchical agglomerative clustering method: which algorithms implement ward’s criterion?. J Classif.

[43] Nielsen F, Nielsen F (2016). Hierarchical clustering. Introduction to HPC with MPI for Data Science.

[44] van der Maaten L, Hinton G (2008). Viualizing data using t-SNE. J Mach Learn Res.

[45] Ke G, Meng Q, Finley T, Wang T, Chen W, Ma W, Ye Q, Liu TY (2017). LightGBM: a highly efficient gradient boosting decision tree. Proceedings of the 31st International Conference on Neural Information Processing Systems.

[46] Prokhorenkova L, Gusev G, Vorobev A, Dorogush AV, Gulin A (2018). CatBoost: unbiased boosting with categorical features. Proceedings of the 32nd International Conference on Neural Information Processing Systems.

[47] Bergstra J, Bengio Y (2012). Random Search for Hyper-Parameter Optimization. J Mach Learn Res.

[48] Chawla NV, Bowyer KW, Hall LO, Kegelmeyer WP (2002). SMOTE: synthetic minority over-sampling technique. J Artif Intell.

[49] Breiman L (2001). Random forests. Mach Learn.

[50] Lundberg SM, Lee SI (2017). A unified approach to interpreting model predictions. Proceedings of the 31st International Conference on Neural Information Processing Systems.

[51] Lundberg SM, Erion G, Chen H, DeGrave A, Prutkin JM, Nair B, Katz R, Himmelfarb J, Bansal N, Lee S (2020). From local explanations to global understanding with explainable AI for trees. Nat Mach Intell.

[52] Virtanen P, Gommers R, Oliphant TE, Haberland M, Reddy T, Cournapeau D, Burovski E, Peterson P, Weckesser W, Bright J, van der Walt SJ, Brett M, Wilson J, Millman KJ, Mayorov N, Nelson AR, Jones E, Kern R, Larson E, Carey CJ, Polat İ, Feng Y, Moore EW, VanderPlas J, Laxalde D, Perktold J, Cimrman R, Henriksen I, Quintero EA, Harris CR, Archibald AM, Ribeiro AH, Pedregosa F, van Mulbregt P (2020). SciPy 1.0: fundamental algorithms for scientific computing in Python. Nat Methods.

[53] Puttonen S, Härmä M, Hublin C (2010). Shift work and cardiovascular disease - pathways from circadian stress to morbidity. Scand J Work Environ Health.

[54] Ritterband LM, Thorndike FP, Ingersoll KS, Lord HR, Gonder-Frederick L, Frederick C, Quigg MS, Cohn WF, Morin CM (2017). Effect of a web-based cognitive behavior therapy for insomnia intervention with 1-year follow-up: a randomized clinical trial. JAMA Psychiatry.

