# Supplementary material for: Personalized Physician-Assisted Sleep Advice for Shift Workers: Algorithm Development and Validation Study
Source: JMIR Form Res. 2025 Apr 1;9:e65000. doi: 10.2196/65000 (PMC12000788; doi:10.2196/65000)
Supplement: Multimedia Appendix 1 [file formative_v9i1e65000_app1.docx]

Supplementary Files

Table S1. Descriptions of behavioral and physiological features used for developing sleep advice prediction models

| Modality | Submodality | Feature | Description |
| --- | --- | --- | --- |
|  |  |  |  |
| **Fitbit** |  |  |  |
|  | Heart Rate  (6 features) | hrmean_mean, hrmean_sd | Mean and SD of average daily heart rate across previous 4 days |
|  |  | hrstd_mean, hrstd_sd | Mean and SD of daily heart rate SD across previous 4 days |
|  |  | hrentropy_mean, hrentropy_sd | Mean and SD of heart rate sample entropy across previous 4 days |
|  | Steps (6 features) | steps_mean, steps_sd | Mean and SD of daily step counts across previous 4 days |
|  |  | duration_entropy_non_step_mean, duration_entropy_non_step_sd | Mean and SD of information entropy of continuous active segments across previous 4 days |
|  |  | duration_entropy_step_mean, duration_entropy_step_sd | Mean and SD of information entropy of continuous stationary segments across previous 4 days |
| **Morning Survey** |  |  |  |
|  | Sleep (33 features) |  |  |
|  |  | sleep_prev_24_mean, sleep_prev_24_sd | Mean and SD of the binary indicator of sleep in the past 24 hours across previous 4 days |
|  |  | wake_natural_mean, wake_natural_sd | Mean and SD of the binary indicator of waking up naturally across previous 4 days |
|  |  | wake_alarm_mean,  wake_alarm_sd | Mean and SD of the binary indicator of waking up by alarms across previous 4 days |
|  |  | wake_other_mean, wake_other_sd | Mean and SD of the binary indicator of waking up by other means across previous 4 days |
|  |  | time_to_asleep_mean, time_to_asleep_sd | Mean and SD of the time taken to sleep across previous 4 days Feature value encoding: 1-5 minutes: 1 6-15 minutes: 2 16-30 minutes: 3 31-60 minutes: 4 more than 61 minutes: 5 |
|  |  | phone_prior_sleep_mean, phone_prior_sleep_sd | Mean and SD of the amount of time prior to sleep that participants were using phones across previous 4 days Feature value encoding: 0-5 minutes: 1 5-15 minutes: 2 15-30 minutes: 3 30-60 minutes: 4 more than 60 minutes: 5 |
|  |  | brightness_sleep_mean, brightness_sleep_sd | Mean and SD of brightness level of sleep environment across previous 4 days |
|  |  | nap_count_mean, nap_count_sd | Mean and SD of nap counts across previous 4 days |
|  |  | deep_sleep_mean deep_sleep_sd | Mean and SD of answers to the statement "I slept soundly" from strongly disagree (1) to strongly agree (5) across previous 4 days |
|  |  | immediate_sleep_mean immediate_sleep_sd | Mean and SD of answers to the statement "I fell asleep immediately" from strongly disagree (1) to strongly agree (5) across previous 4 days |
|  |  | fatigue_recover_mean, fatigue_recover_sd | Mean and SD of answers to the statement "I was able to recover from fatigue" from strongly disagree (1) to strongly agree (5) across previous 4 days |
|  |  | mid_awake_mean, mid_awake_sd | Mean and SD of answers to the statement "I didn't wake up in the middle of sleep" from strongly disagree (1) to strongly agree (5) across previous 4 days |
|  |  | sleep_satisfy_mean, sleep_satisfy_sd | Mean and SD of answers to the statement "I was satisfied with sleep" from strongly disagree (1) to strongly agree (5) across previous 4 days |
|  |  | sleep_regularity_3, sleep_regularity_5, sleep_regularity_7 | Sleep regularity index with sliding windows of 3, 5, and 7 days |
|  |  | sleep_duration_mean, sleep_duration_sd | Mean and SD of main sleep duration across previous 4 days |
|  |  | nap_duration_mean, nap_duration_sd | Mean and SD of nap duration across previous 4 days |
|  | Wellbeing (12 features) |  |  |
|  |  | alertness_morn_mean, alertness_morn_sd | Mean and SD of morning alertness from 0-100 (most positive) across previous 4 days |
|  |  | happiness_morn_mean, happiness_morn_sd | Mean and SD of morning happiness from 0-100 (most positive) across previous 4 days |
|  |  | energy_morn_mean, energy_morn_sd | Mean and SD of morning energy from 0-100 (most positive) across previous 4 days |
|  |  | health_morn_mean, health_morn_sd | Mean and SD of morning health from 0-100 (most positive) across previous 4 days |
|  |  | calmness_morn_mean, calmness_morn_sd | Mean and SD of morning calmness from 0-100 (most positive) across previous 4 days |
|  |  | sleepiness_morn_mean, sleepiness_morn_sd | Mean and SD of morning sleepiness from strongly awake (1) to strongly sleepy (9) across previous 4 days |
|  | Behavior  (8 features) |  |  |
|  |  | caffeine_amount_mean, caffeine_amount_sd | Mean and SD of daily caffeine intake across previous 4 days |
|  |  | alcohol_amount_mean, alcohol_amount_sd | Mean and SD of daily alcohol intake across previous 4 days |
|  |  | caffeine_before_sleep_mean,  caffeine_before_sleep_sd | Mean and SD of duration between last caffeine intake and sleep across previous 4 days |
|  |  | bath_before_sleep_mean,  bath_before_sleep_sd | Mean and SD of duration between bath and sleep across previous 4 days |
| **Evening Survey** |  |  |  |
|  | Work (32 features) | worktime_prev2_duration_mean,  worktime_prev2_duration_sd, worktime_prev2_duration1_mean,  worktime_prev2_duration1_sd, worktime_prev2_duration2_mean,  worktime_prev2_duration2_sd, worktime_prev2_duration3_mean,  worktime_prev2_duration3_sd | Mean and SD of work hours of the day before yesterday for the entire day, 0:00-8:00, 8:00-16:00, and 16:00-24:00 summarized across previous 4 days |
|  |  | worktime_prev1_duration_mean,  worktime_prev1_duration_sd, worktime_prev1_duration1_mean,  worktime_prev1_duration1_sd, worktime_prev1_duration2_mean,  worktime_prev1_duration2_sd, worktime_prev1_duration3_mean,  worktime_prev1_duration3_sd | Mean and SD of work hours of yesterday for the entire day, 0:00-8:00, 8:00-16:00, and 16:00-24:00 summarized across previous 4 days |
|  |  | worktime_today_duration_mean,  worktime_today_duration_sd, worktime_today_duration1_mean,  worktime_today_duration1_sd, worktime_today_duration2_mean,  worktime_today_duration2_sd, worktime_today_duration3_mean,  worktime_today_duration3_sd | Mean and SD of work hours today for the entire day, 0:00-8:00, 8:00-16:00, and 16:00-24:00 summarized across previous 4 days |
|  |  | extrawork_activities_mean extrawork_activities_sd | Mean and SD of the binary indicator of activities other than work outside homes across previous 4 days |
|  |  | nap_duration_in_work_mean,  nap_duration_in_work_sd | Mean and SD of nap duration during work hours across previous 4 days |
|  |  | gap_work_sleep_mean,  gap_work_sleep_sd | Mean and SD of the minimum duration between the end of shifts and following sleep periods across previous 4 days |
|  |  | gap_sleep_work_mean,  gap_sleep_work_sd | Mean and SD of the minimum duration between the last sleep periods and the start of shifts across previous 4 days |
|  | Wellbeing (18 features) |  |  |
|  |  | alertness_eve_mean alertness_eve_sd | Mean and SD of self-reported evening alertness from 0-100 (most positive) across previous 4 days |
|  |  | happiness_eve_mean, happiness_eve_sd | Mean and SD of self-reported evening happiness from 0-100 (most positive) across previous 4 days |
|  |  | energy_eve_mean energy_eve_sd | Mean and SD of self-reported evening energy from 0-100 (most positive) across previous 4 days |
|  |  | health_eve_mean, health_eve_sd | Mean and SD of self-reported evening health from 0-100 (most positive) across previous 4 days |
|  |  | calmness_eve_mean, calmness_eve_sd | Mean and SD of self-reported evening calmness from 0-100 (most positive) across previous 4 days |
|  |  | sleepiness_now_eve_mean, sleepiness_now_eve_sd | Mean and SD of self-reported evening sleepiness from strongly awake (1) to strongly sleepy (9) across previous 4 days |
|  |  | sleepiness_daytime_mean, sleepiness_daytime_sd | Mean and SD of self-reported sleepiness during the day from strongly sleepy (1) to strongly awake (5) across previous 4 days |
|  |  | stress_mean, stress_sd | Mean and SD of self-reported stress levels across previous 4 days |
|  |  | tiredness_mean, tiredness_sd | Mean and SD of self-reported tiredness levels across previous 4 days |
| Advice Response |  | advice_1_response,  advice_2_response,  advice_3_response,  advice_4_response,  advice_5_response,  advice_6_response,  advice_7_response,  advice_8_response,  advice_9_response,  advice_10_response,  advice_11_response,  advice_12_response,  advice_13_response,  advice_14_response,  advice_15_response,  advice_16_response,  advice_17_response,  advice_18_response,  advice_19_response,  advice_20_response,  advice_21_response,  advice_22_response,  advice_23_response | Binary indicator of the response to the previous advice message given, where 0 means no response or “difficult to follow” and 1 means “eager to follow.” For example, if the previous message is advice 20 with response “eager to follow”, then advice_20_response will be 1 and all other columns will be 0. |

Table S2. Sizes and profiles of clusters and subclusters from hierarchical clustering of daily features

| Cluster  ID | Cluster size | Cluster profile | Subcluster  ID | Subcluster size | Subcluster profile |
| --- | --- | --- | --- | --- | --- |
| 0 | 602 | Fitbit: above average heart rate SD, above average step counts, higher variability of active behaviors  Sleep: below average time to fall asleep, above average brightness during sleep, high sleep quality (deep sleep, asleep immediately, fatigue recovery, sleep satisfaction), around 50% waking up naturally and waking up by alarm and other means either takes around 25%, longest average sleep duration around 470 minutes  Work: slightly more proportion with shifts the day before yesterday, slightly more proportion with shifts yesterday, majorly shifts today   Wellbeing: above average alertness in the evening, more awake in the evening, above average alertness and health in the morning  Miscellany: above average daily caffeine consumption | c | 144 | 90% waking up by other means, above average proportion with activities other than work. |
|  |  |  | e | 66 | Shortest time to fall asleep, shortest time prior to sleep with phone usage, highest brightness in sleep. Overtime shift of 10-11 hours the day before yesterday, overtime shift of 9-10 hours yesterday, overtime shift of 11-12 hours today. Highest daily caffeine consumption. |
|  |  |  | f | 126 | Above average proportion of waking up naturally, sleep duration above 500 minutes. Majorly shifts the day before yesterday, majorly shifts today (majorly afternoon shift) |
|  |  |  | g | 159 | Above average proportion of waking up naturally. Majorly shifts today (majorly afternoon shift). Above average alcohol consumption. |
|  |  |  | m | 107 | Sleep duration above 500 minutes, majorly no naps. Majorly shifts yesterday (majorly day shifts). |
| 1 | 416 | Fitbit: below average step counts, higher variability of stationary behaviors, lower variability of active behaviors Sleep: above average brightness during sleep, high sleep quality (deep sleep, asleep immediately, fatigue recovery, awake in the middle of sleep, sleep satisfaction), around 60% waking up naturally and 40% by alarm Work: slightly more proportion with shifts the day before yesterday, slightly more proportion with shifts yesterday , majorly no shifts today  Wellbeing: high wellbeing (alertness, happiness, energy, health, calmness) in the evening, less stressed and tired in the evening, high wellbeing (alertness, happiness, energy, health, calmness) in the morning, more awake in the morning  Miscellany: average caffeine consumption | a | 210 | Above average alcohol consumption. Around half with shifts the day before yesterday, majorly no shifts yesterday. |
|  |  |  | b | 206 | Sleep duration under 400 minutes. Majorly shifts the day before yesterday (majorly afternoon shift), majorly shifts yesterday (majorly afternoon shifts). |
| 2 | 217 | Fitbit: below average step counts  Sleep: above average daily nap counts, 50% waking up naturally and 50% waking up by alarm, longest average nap duration of around 160 minutes  Work: almost all shifts the day before yesterday, almost all shifts yesterday (almost all midnight shifts), around half with shifts today   Wellbeing: less stressed in the evening  Miscellany: average caffeine consumption | d | 154 | Longest nap duration around 200 minutes  Higher wellbeing scores(sleepiness during the day, evening alertness, energy, health, tiredness, morning alertness, happiness, energy, health, calmness, sleepiness) and better sleep quality than subcluster d   Majorly no shifts today |
|  |  |  | k | 63 | Sleep duration under 400 minutes, below average sleep quality (deep sleep, fatigue recovery, asleep immediately, sleep satisfaction)  Almost all day shifts the day before yesterday, all most all midnight shifts today  No alcohol consumption |
| 3 | 195 | Sleep: much shorter average sleep duration of around 220 minutes and the lowest sleep quality  Work: day shifts the day before and midnight shifts on the day for almost all samples.  Wellbeing: sleepiest during the day, low wellbeing (alertness, happiness, energy, health, calmness) in the evening and morning  Miscellany: low caffeine and alcohol intake | h | N/A |  |
| 4 | 2 | Fitbit: high average heart rate  Sleep: no sleep in previous 24 hours  Work: no midnight shifts the day before yesterday  Wellbeing: lowest wellbeing (alertness, energy, health) but highest happiness in the evening, sleepiest in the evening, most stressed and tired in the evening, lowest wellbeing (alertness, happiness, energy, health, calmness) in the morning, sleepiest in the morning  Miscellany: lowest caffeine consumption, no alcohol consumption | j | N/A |  |
| 5 | 203 | Fitbit: above average heart rate SD, below average heart rate entropy, above average step counts, lower variability of stationary behaviors, higher variability of active behaviors  Sleep: above average time to fall asleep, below average brightness during sleep, around 90% waking up by alarm  Work: majorly no activities other than work, slightly more proportion with shifts the day before yesterday, majorly no shifts yesterday , almost all shifts today (almost all day shifts)  Wellbeing: more awake during the days, low wellbeing (alertness, happiness, energy, health, calmness) in the evening, much more stressed and tired in the evening, sleepy in the evening, low wellbeing (alertness, happiness, energy, calmness) in the morning, sleepy in the morning  Miscellany: average caffeine consumption | i | 151 | Highest heart rate SD, only cluster with more than 10000 daily step counts |
|  |  |  | l | 52 | Lowest average heart rate, only cluster with fewer than 2000 daily step counts  Sleep duration under 400 minutes |

Table S3. Distributions of positive and negative advice labels for both the training and test data under user-dependent and independent settings

| Setting | Message ID | Split | Negative Count | Positive Count | Negative-to-Positive Ratio |
| --- | --- | --- | --- | --- | --- |
| **Dependent** |  |  |  |  |  |
|  | 4 | Train | 461 | 46 | 10.02 |
|  |  | Test | 214 | 27 | 7.93 |
|  | 7 | Train | 393 | 114 | 3.45 |
|  |  | Test | 202 | 39 | 5.18 |
|  | 12 | Train | 469 | 38 | 12.34 |
|  |  | Test | 215 | 26 | 8.27 |
|  | 14 | Train | 458 | 49 | 9.35 |
|  |  | Test | 215 | 26 | 8.27 |
|  | 15 | Train | 449 | 58 | 7.74 |
|  |  | Test | 199 | 42 | 4.74 |
|  | 20 | Train | 273 | 234 | 1.17 |
|  |  | Test | 107 | 134 | 0.80 |
|  | 21 | Train | 354 | 153 | 2.31 |
|  |  | Test | 189 | 52 | 3.63 |
| **Independent** |  |  |  |  |  |
|  | 4 | Train | 469 | 52 | 9.02 |
|  |  | Test | 206 | 21 | 9.81 |
|  | 7 | Train | 401 | 120 | 3.34 |
|  |  | Test | 194 | 33 | 5.88 |
|  | 12 | Train | 464 | 57 | 8.14 |
|  |  | Test | 220 | 7 | 31.43 |
|  | 14 | Train | 457 | 64 | 7.14 |
|  |  | Test | 216 | 11 | 19.64 |
|  | 15 | Train | 434 | 87 | 4.99 |
|  |  | Test | 214 | 13 | 16.46 |
|  | 20 | Train | 267 | 254 | 1.05 |
|  |  | Test | 113 | 114 | 0.99 |
|  | 21 | Train | 369 | 152 | 2.43 |
|  |  | Test | 174 | 53 | 3.28 |

Table S4. Advice prediction sensitivity, specificity, precision and F1 of the ensemble RF classifiers, obtained under different data balancing approaches (none, random oversampling, SMOTE) and dataset split settings (user-dependent, user-independent). Ensemble classifiers were constructed by majority voting across 9 RF classifiers trained with different random seeds. The decision threshold for each classifier was optimized to maximize the F1 score. The F1 baseline from always predicting the positive class is also listed.

| Setting | Message ID | Balancing Method | Sensitivity | Specificity | Precision | F1 | F1 baseline (always positive) |
| --- | --- | --- | --- | --- | --- | --- | --- |
| **Dependent** |  |  |  |  |  |  |  |
|  | 4 | None | 0.33 | 0.92 | 0.35 | 0.34 | 0.20 |
|  |  | Random Oversampling | 0.37 | 0.79 | 0.19 | 0.25 |  |
|  |  | SMOTE | 0.41 | 0.86 | 0.27 | 0.32 |  |
|  | 7 | None | 0.67 | 0.84 | 0.45 | 0.54 | 0.28 |
|  |  | Random Oversampling | 0.74 | 0.80 | 0.41 | 0.53 |  |
|  |  | SMOTE | 0.69 | 0.81 | 0.42 | 0.52 |  |
|  | 12 | None | 0.50 | 0.83 | 0.27 | 0.35 | 0.19 |
|  |  | Random Oversampling | 0.31 | 0.92 | 0.32 | 0.31 |  |
|  |  | SMOTE | 0.31 | 0.89 | 0.25 | 0.28 |  |
|  | 14 | None | 0.62 | 0.72 | 0.21 | 0.31 | 0.19 |
|  |  | Random Oversampling | 0.58 | 0.81 | 0.27 | 0.37 |  |
|  |  | SMOTE | 0.65 | 0.70 | 0.21 | 0.31 |  |
|  | 15 | None | 0.71 | 0.53 | 0.24 | 0.36 | 0.30 |
|  |  | Random Oversampling | 0.55 | 0.72 | 0.29 | 0.38 |  |
|  |  | SMOTE | 0.31 | 0.83 | 0.28 | 0.30 |  |
|  | 20 | None | 0.85 | 0.45 | 0.66 | 0.74 | 0.71 |
|  |  | Random Oversampling | 0.82 | 0.52 | 0.68 | 0.75 |  |
|  |  | SMOTE | 0.84 | 0.50 | 0.67 | 0.75 |  |
|  | 21 | None | 0.87 | 0.38 | 0.28 | 0.42 | 0.35 |
|  |  | Random Oversampling | 0.87 | 0.33 | 0.26 | 0.40 |  |
|  |  | SMOTE | 0.77 | 0.57 | 0.33 | 0.46 |  |
| **Independent** |  |  |  |  |  |  |  |
|  | 4 | None | 0.95 | 0.54 | 0.17 | 0.29 | 0.17 |
|  |  | Random Oversampling | 1.00 | 0.46 | 0.16 | 0.27 |  |
|  |  | SMOTE | 1.00 | 0.33 | 0.13 | 0.23 |  |
|  | 7 | None | 0.45 | 0.97 | 0.71 | 0.56 | 0.25 |
|  |  | Random Oversampling | 0.45 | 0.97 | 0.71 | 0.56 |  |
|  |  | SMOTE | 0.48 | 0.97 | 0.73 | 0.58 |  |
|  | 12 | None | 0.57 | 0.69 | 0.05 | 0.10 | 0.06 |
|  |  | Random Oversampling | 0.71 | 0.64 | 0.06 | 0.11 |  |
|  |  | SMOTE | 0.43 | 0.80 | 0.06 | 0.11 |  |
|  | 14 | None | 0.73 | 0.63 | 0.09 | 0.16 | 0.09 |
|  |  | Random Oversampling | 0.64 | 0.60 | 0.08 | 0.13 |  |
|  |  | SMOTE | 0.55 | 0.61 | 0.07 | 0.12 |  |
|  | 15 | None | 0.85 | 0.29 | 0.07 | 0.12 | 0.11 |
|  |  | Random Oversampling | 0.85 | 0.36 | 0.07 | 0.14 |  |
|  |  | SMOTE | 0.77 | 0.48 | 0.08 | 0.15 |  |
|  | 20 | None | 0.82 | 0.47 | 0.61 | 0.70 | 0.67 |
|  |  | Random Oversampling | 0.82 | 0.42 | 0.58 | 0.68 |  |
|  |  | SMOTE | 0.82 | 0.46 | 0.61 | 0.70 |  |
|  | 21 | None | 0.79 | 0.40 | 0.29 | 0.42 | 0.38 |
|  |  | Random Oversampling | 0.81 | 0.44 | 0.31 | 0.45 |  |
|  |  | SMOTE | 0.74 | 0.49 | 0.30 | 0.43 |  |

Table S5. Advice prediction sensitivity, specificity, precision and F1 of the ensemble LGBM classifiers, obtained under different data balancing approaches (none, random oversampling, SMOTE) and dataset split settings (user-dependent, user-independent). Ensemble classifiers were constructed by majority voting across 9 LGBM classifiers trained with different random seeds. The decision threshold for each classifier was optimized to maximize the F1 score. The F1 baseline from always predicting the positive class is also listed.

| Setting | Message ID | Balancing Method | Sensitivity | Specificity | Precision | F1 | F1 baseline (always positive) |
| --- | --- | --- | --- | --- | --- | --- | --- |
| **Dependent** |  |  |  |  |  |  |  |
|  | 4 | None | 0.56 | 0.86 | 0.34 | 0.42 | 0.20 |
|  |  | Random Oversampling | 0.11 | 0.92 | 0.14 | 0.12 |  |
|  |  | SMOTE | 0.19 | 0.88 | 0.16 | 0.17 |  |
|  | 7 | None | 0.72 | 0.85 | 0.48 | 0.58 | 0.28 |
|  |  | Random Oversampling | 0.67 | 0.84 | 0.44 | 0.53 |  |
|  |  | SMOTE | 0.62 | 0.87 | 0.47 | 0.53 |  |
|  | 12 | None | 0.15 | 0.96 | 0.31 | 0.21 | 0.19 |
|  |  | Random Oversampling | 0.12 | 0.96 | 0.27 | 0.16 |  |
|  |  | SMOTE | 0.08 | 0.98 | 0.33 | 0.12 |  |
|  | 14 | None | 0.42 | 0.86 | 0.27 | 0.33 | 0.19 |
|  |  | Random Oversampling | 0.27 | 0.87 | 0.19 | 0.23 |  |
|  |  | SMOTE | 0.50 | 0.81 | 0.25 | 0.33 |  |
|  | 15 | None | 0.86 | 0.32 | 0.21 | 0.34 | 0.30 |
|  |  | Random Oversampling | 0.90 | 0.32 | 0.22 | 0.35 |  |
|  |  | SMOTE | 0.33 | 0.77 | 0.23 | 0.27 |  |
|  | 20 | None | 0.84 | 0.39 | 0.63 | 0.72 | 0.71 |
|  |  | Random Oversampling | 0.84 | 0.41 | 0.64 | 0.73 |  |
|  |  | SMOTE | 0.84 | 0.40 | 0.64 | 0.72 |  |
|  | 21 | None | 0.77 | 0.51 | 0.30 | 0.43 | 0.35 |
|  |  | Random Oversampling | 0.83 | 0.40 | 0.27 | 0.41 |  |
|  |  | SMOTE | 0.85 | 0.50 | 0.32 | 0.46 |  |
| **Independent** |  |  |  |  |  |  |  |
|  | 4 | None | 1.00 | 0.44 | 0.15 | 0.27 | 0.17 |
|  |  | Random Oversampling | 0.86 | 0.49 | 0.15 | 0.25 |  |
|  |  | SMOTE | 0.52 | 0.53 | 0.10 | 0.17 |  |
|  | 7 | None | 0.45 | 0.97 | 0.71 | 0.56 | 0.25 |
|  |  | Random Oversampling | 0.45 | 0.97 | 0.71 | 0.56 |  |
|  |  | SMOTE | 0.52 | 0.97 | 0.74 | 0.61 |  |
|  | 12 | None | 0.86 | 0.65 | 0.07 | 0.13 | 0.06 |
|  |  | Random Oversampling | 1.00 | 0.60 | 0.07 | 0.14 |  |
|  |  | SMOTE | 0.43 | 0.81 | 0.07 | 0.12 |  |
|  | 14 | None | 0.64 | 0.61 | 0.08 | 0.14 | 0.09 |
|  |  | Random Oversampling | 0.73 | 0.40 | 0.06 | 0.11 |  |
|  |  | SMOTE | 0.18 | 0.89 | 0.08 | 0.11 |  |
|  | 15 | None | 0.85 | 0.32 | 0.07 | 0.13 | 0.11 |
|  |  | Random Oversampling | 0.62 | 0.36 | 0.06 | 0.10 |  |
|  |  | SMOTE | 0.69 | 0.46 | 0.07 | 0.13 |  |
|  | 20 | None | 0.78 | 0.50 | 0.61 | 0.69 | 0.67 |
|  |  | Random Oversampling | 0.80 | 0.49 | 0.61 | 0.69 |  |
|  |  | SMOTE | 0.78 | 0.46 | 0.59 | 0.67 |  |
|  | 21 | None | 0.77 | 0.44 | 0.29 | 0.43 | 0.38 |
|  |  | Random Oversampling | 0.77 | 0.46 | 0.30 | 0.44 |  |
|  |  | SMOTE | 0.79 | 0.45 | 0.31 | 0.44 |  |

Table S6. Advice prediction sensitivity, specificity, precision and F1 of the ensemble CatBoost classifiers, obtained under different data balancing approaches (none, random oversampling, SMOTE) and dataset split settings (user-dependent, user-independent). Ensemble classifiers were constructed by majority voting across 9 CatBoost classifiers trained with different random seeds. The decision threshold for each classifier was optimized to maximize the F1 score. The F1 baseline from always predicting the positive class is also listed.

| Setting | Message ID | Balancing Method | Sensitivity | Specificity | Precision | F1 | F1 baseline (always positive) |
| --- | --- | --- | --- | --- | --- | --- | --- |
| **Dependent** |  |  |  |  |  |  |  |
|  | 4 | None | 0.41 | 0.90 | 0.34 | 0.37 | 0.20 |
|  |  | Random Oversampling | 0.22 | 0.90 | 0.21 | 0.22 |  |
|  |  | SMOTE | 0.26 | 0.91 | 0.26 | 0.26 |  |
|  | 7 | None | 0.69 | 0.87 | 0.50 | 0.58 | 0.28 |
|  |  | Random Oversampling | 0.69 | 0.88 | 0.52 | 0.59 |  |
|  |  | SMOTE | 0.72 | 0.85 | 0.47 | 0.57 |  |
|  | 12 | None | 0.12 | 0.99 | 0.60 | 0.19 | 0.19 |
|  |  | Random Oversampling | 0.23 | 0.92 | 0.26 | 0.24 |  |
|  |  | SMOTE | 0.19 | 0.93 | 0.26 | 0.22 |  |
|  | 14 | None | 0.85 | 0.65 | 0.23 | 0.36 | 0.19 |
|  |  | Random Oversampling | 0.77 | 0.64 | 0.21 | 0.33 |  |
|  |  | SMOTE | 0.54 | 0.77 | 0.22 | 0.31 |  |
|  | 15 | None | 0.52 | 0.68 | 0.26 | 0.34 | 0.30 |
|  |  | Random Oversampling | 0.55 | 0.61 | 0.23 | 0.32 |  |
|  |  | SMOTE | 0.29 | 0.81 | 0.24 | 0.26 |  |
|  | 20 | None | 0.87 | 0.38 | 0.64 | 0.74 | 0.71 |
|  |  | Random Oversampling | 0.88 | 0.36 | 0.63 | 0.74 |  |
|  |  | SMOTE | 0.89 | 0.34 | 0.63 | 0.73 |  |
|  | 21 | None | 0.81 | 0.58 | 0.34 | 0.48 | 0.35 |
|  |  | Random Oversampling | 0.85 | 0.48 | 0.31 | 0.45 |  |
|  |  | SMOTE | 0.77 | 0.57 | 0.33 | 0.46 |  |
| **Independent** |  |  |  |  |  |  |  |
|  | 4 | None | 0.90 | 0.52 | 0.16 | 0.28 | 0.17 |
|  |  | Random Oversampling | 0.76 | 0.43 | 0.12 | 0.21 |  |
|  |  | SMOTE | 0.57 | 0.56 | 0.12 | 0.19 |  |
|  | 7 | None | 0.42 | 0.97 | 0.74 | 0.54 | 0.25 |
|  |  | Random Oversampling | 0.61 | 0.96 | 0.71 | 0.66 |  |
|  |  | SMOTE | 0.73 | 0.93 | 0.63 | 0.68 |  |
|  | 12 | None | 0.43 | 0.62 | 0.03 | 0.06 | 0.06 |
|  |  | Random Oversampling | 1.00 | 0.58 | 0.07 | 0.13 |  |
|  |  | SMOTE | 0.57 | 0.75 | 0.07 | 0.12 |  |
|  | 14 | None | 0.91 | 0.68 | 0.12 | 0.22 | 0.09 |
|  |  | Random Oversampling | 0.64 | 0.57 | 0.07 | 0.13 |  |
|  |  | SMOTE | 0.36 | 0.80 | 0.08 | 0.14 |  |
|  | 15 | None | 0.85 | 0.19 | 0.06 | 0.11 | 0.11 |
|  |  | Random Oversampling | 0.77 | 0.21 | 0.06 | 0.10 |  |
|  |  | SMOTE | 0.69 | 0.30 | 0.06 | 0.11 |  |
|  | 20 | None | 0.75 | 0.51 | 0.61 | 0.67 | 0.67 |
|  |  | Random Oversampling | 0.75 | 0.53 | 0.62 | 0.68 |  |
|  |  | SMOTE | 0.76 | 0.51 | 0.61 | 0.68 |  |
|  | 21 | None | 0.92 | 0.15 | 0.25 | 0.39 | 0.38 |
|  |  | Random Oversampling | 0.92 | 0.19 | 0.26 | 0.40 |  |
|  |  | SMOTE | 0.85 | 0.32 | 0.27 | 0.41 |  |


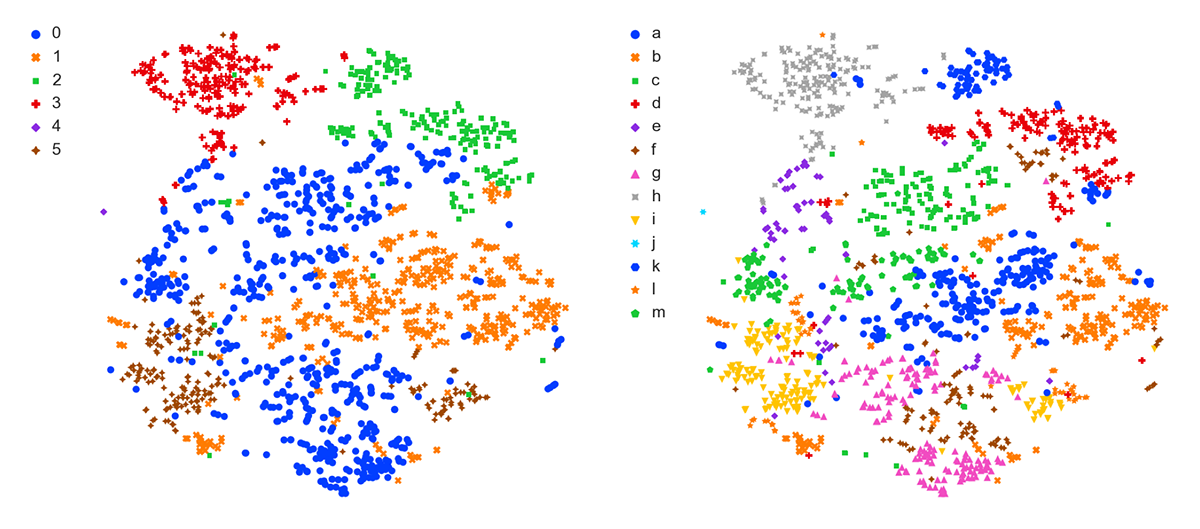


Figure S1: Clusters and subclusters from hierarchical clustering of daily features overlaid on t-SNE plots obtained from the same data used for clustering. Principal components that explained 95% of total variance were used for t-SNE. Plot on the left is overlaid with the 6 clusters identified by distance threshold 52.24 and plot on the right is overlaid with the 13 subclusters identified by threshold 31 in the dendrogram. Same colors are used to label the 6 clusters in the dendrogram and the plot on the left.


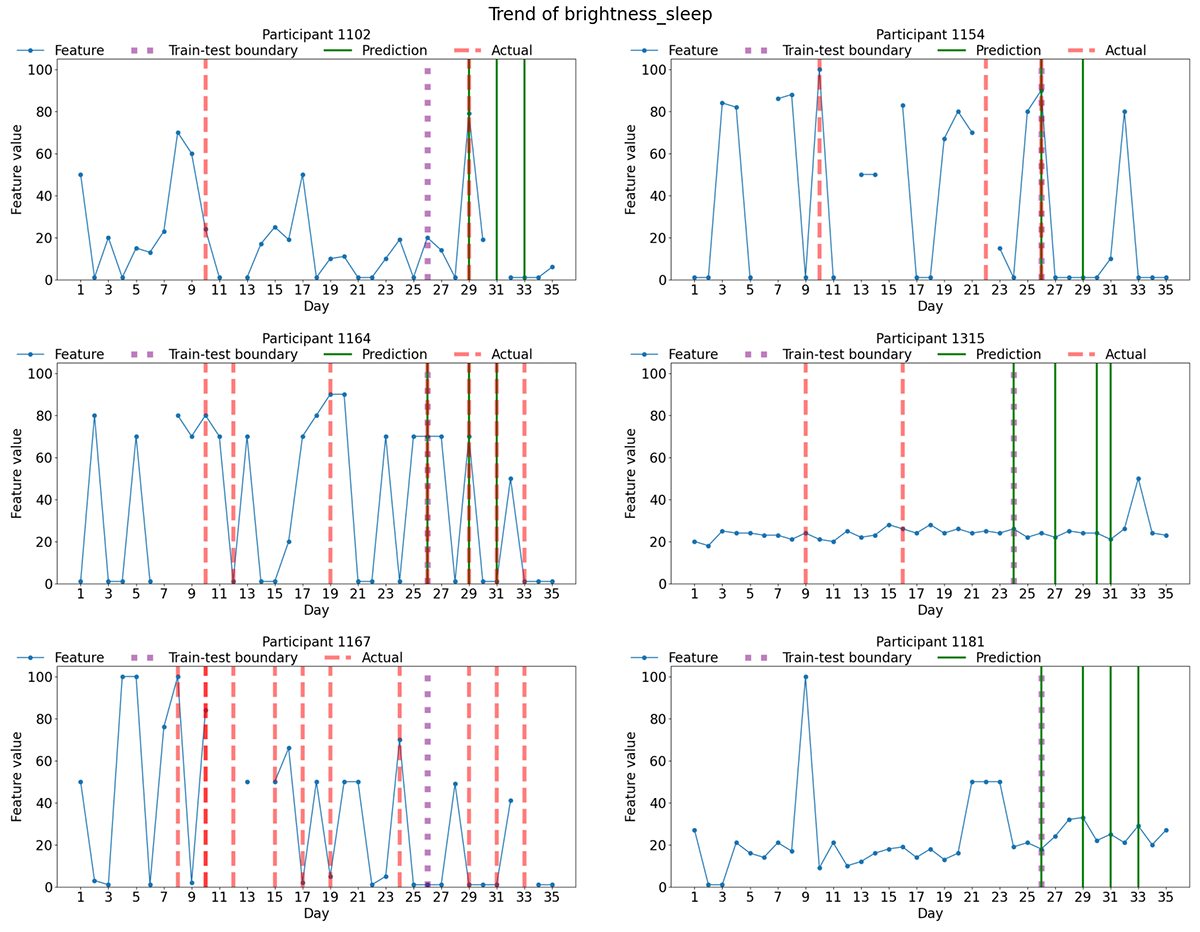


Figure S2: Trends of feature “brightness level of sleep environment” and predictions of message 7 (Darken the bedroom when you go to bed.) under the user-dependent setting for participants 1102, 1154, 1164, 1315, 1167, and 1181. The blue solid line represents available feature values across all days of a participant’s enrollment. The green solid line marks dates with predicted selections of the message, while actual selections are marked by the red dashed line. The purple dashed line corresponds to the boundary between training and testing data such that dates earlier than the boundary belongs to the training data.
